# Electrocatalytic Hydrogenation of Bioinhibiting Aromatics to Bioavailable Aliphatics for Wastewater Denitrification Enhancement

**DOI:** 10.1021/acs.est.5c00950

**Published:** 2025-07-28

**Authors:** Shan Shao, Shicong Du, Asma Batool, Yichun Lu, Kwun Hin Fung, Shuquan Huang, Yangxin Jin, Shengqin Liu, Qi Zhu, Yuhe He, Alicia Kyoungjin An, Patrick K. H. Lee, Jason Chun-Ho Lam

**Affiliations:** † School of Energy and Environment and State Key Laboratory of Marine Environmental Health, 53025 City University of Hong Kong, Kowloon Tong 999077, Hong Kong SAR, China; ‡ Department of Chemical and Biological Engineering, 121835 Hong Kong University of Science and Technology, Clear Water Bay, Kowloon 999077, Hong Kong SAR, China; § Faculty of Chemical Engineering, 47910 Kunming University of Science and Technology, Kunming, Yunnan 650500, China

**Keywords:** Ru/ACC, electrochemical hydrogenation (ECH), refractory organics, wastewater, denitrification

## Abstract

Biological denitrification is a key process for nitrogen removal in wastewater, enabling the conversion of nitrate (NO_3_^–^) to nitrogen gas (N_2_). However, this process can be inhibited in the presence of aromatic substrates. Conventional advanced oxidation protocols involve the mineralization of aromatics, but the widespread adoption of such protocols is limited by high energy and chemical requirements. In this study, we present a novel electrochemical hydrogenation (ECH) protocol to transform bioinhibiting aromatics into bioavailable aliphatic products, thereby promoting biologically driven denitrification. A highly active carbon-supported ruthenium electrode was designed to convert various unsaturated, aromatic, and halogenated aromatic pollutants into aliphatic products, achieving conversion efficiencies of 66.0%–100%. After 5 day incubation, the saturated products enhanced the denitrification rate by up to 614.0%. The relative abundance of denitrifiers in the sequencing batch reactor landfill sludge increased, confirming that the aliphatic products from ECH treatment could enhance bioavailability. This work demonstrates the integration of electrochemical and biological catalysis, valorizing pollutants into biologically useful products that can improve denitrification, especially in existing high-organic-load wastewater treatment networks.

## Graphical abstract

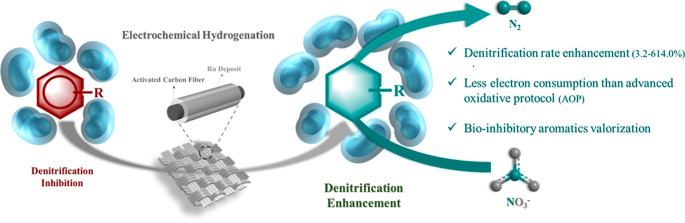

## 1 Introduction

An efficient wastewater treatment scheme is vital for maintaining effective and sustainable wastewater management. The presence of nitrogen in wastewater can pose significant environmental risks if not managed properly.^1–3^ The most common method for removing ammoniacal nitrogen involves a sequencing batch reactor (SBR), where biological nitrification and denitrification alternate in cycles to convert ammonia (NH_3_) to nitrate (NO_3_^–^) aerobically, followed by the conversion of NO_3_^–^ to nitrogen gas (N_2_) in an anoxic environment using dissolved organics as biological electron donors.^4^ However, not all dissolved organics are conducive to biological processes. Many studies have discussed the inhibitory effects of certain unsaturated substrates on biological activity, particularly denitrification. Aromatic compounds, in particular, are highly resistant to biodegradation due to the stability provided by ring resonance.^5^ Their presence can inhibit microbial activities^6^ and significantly alter microbial community composition and diversity.^7^ For example, Ramos et al. reported that phenolic, nitro, and halogen-containing aromatics can inhibit biological treatment.^8^ Some aromatic substrates, like guaiacol,^9^ are susceptible to oxidative condensation, forming insoluble macromolecules that reduce their bioavailability. Halogenated aromatics can even exhibit immediate toxicity.^10^ With expanding industrial activities and urbanization, the prevalence of these organic pollutants is expected to grow in urban and industrial wastewater.^11,12^ Consequently, the growing presence of aromatics poses a significant threat to denitrification efficiency.

In recent years, various technologies have been developed to remove aromatic pollutants from wastewater. Among the most common methods are advanced oxidation protocols (AOPs), which are known as effective pretreatment methods for removing organic contaminants in wastewater^13,14^ by generating reactive oxygen species such as hydroxyl radicals (^•^OH), superoxide radicals (^•^O_2_^–^), and sulfate radicals (^•^SO_4_^–^)^15^ to trigger oxidative mineralization of organic contaminants.^16^ Although AOPs have been established for various catalytic contexts, including photo-, electro-, chemo-, and sono-catalysis, they involve the oxidative mineralization of organics, which is energy- and chemical-intensive and may necessitate downstream oxidant removal to ensure complete mineralization.^17,18^ Moreover, in chlorinated environments, incomplete mineralization could lead to the generation of highly toxic secondary pollutants.^19^

This study proposes an electrochemical hydrogenation (ECH) approach as a novel pretreatment strategy to enhance biological denitrification by transforming wastewater aromatic pollutants. Unlike conventional AOPs that rely on oxidative mineralization, ECH leverages reducing equivalents—protons (H^+^) and electrons (e^–^)—from water through electrochemical bias, eliminating the need for auxiliary reagents and reducing costs and risks to downstream biological treatment. Furthermore, ECH treatment provides a more energy-efficient route to manage organic substrates. For example, the complete electrochemical oxidative mineralization of phenol (C_6_H_5_OH) to carbon dioxide (CO_2_) and water (H_2_O) releases 28 electrons (C_6_H_5_OH + 11H_2_O → 6CO_2_ + 28H^+^ + 28e^–^),^20^ whereas the ECH reduction of phenol to bioavailable cyclohexanol requires only 6 electrons. In this study, ruthenium (Ru) was selected as the electrocatalyst for its cost-effectiveness—being only one-third the price of platinum (Pt)^21^—and its effectiveness in the ECH of aromatic and polyunsaturated substrates such as phenols,^22^ halogen-containing organic pollutants,^23^ aldehydes,^24^ ketones,^25^ and furans.^26,27^ This process transforms such substrates into more biologically available products that are useful for denitrifiers, as verified using SBR denitrification sludge. Thus, we demonstrate a novel application of ECH to convert bioinhibiting aromatic substrates into bioavailable aliphatic products, increasing the relative abundance of denitrifying taxa. This represents a synergetic solution to valorizing aromatic compounds and enhancing biological denitrification.

## 2 Materials and Methods

### 2.1 Electrode Preparation

#### 2.1.1 Activated Carbon Cloth (ACC) Pretreatment

The supporting material is low-cost and flexible carbon cloth, after a two-step activation, acid oxidation followed by air calcination to enhance its surface area and durability.^28^ First, the carbon cloth was submerged in a mixture of 98% H_2_SO_4_, 70% HNO_3_, and deionized (DI, all acronyms used in this work were summarized in Table S1) water (1:1:1 v/v %) at 70 °C for 24 h to induce surface chemical oxidation and then calcinated in an air atmosphere at 500 °C for 2 h. The activation process^29^ facilitate it with uniform nanoporous (Figure S1) and superhydrophilic surfaces (Figure S2). The ACC electrodes were rinsed with DI water and immediately used for Ru deposition.

#### 2.1.2 Ruthenium Deposition on ACC (Ru/ACC)

The Ru catalyst was electrochemically deposited onto the ACC electrode through cyclic voltammetry (CV) with a cycling working potential from −0.5 to −0.9 V vs a silver/silver chloride (Ag/AgCl) reference electrode at a sweep rate of 100 mV s^−1^ in 0.5 M H_2_SO_4_ solution with 20 mM ruthenium (III) chloride (RuCl_3_) at 20 ± 1 °C (Figure 1a).^30^ After Ru electrodeposition, the electrode was rinsed with DI water and dried in a vacuum oven at 20 ± 1 °C for 12 h. The resulting electrode was labeled as the Ru/ACC electrode.

**Figure 1:**
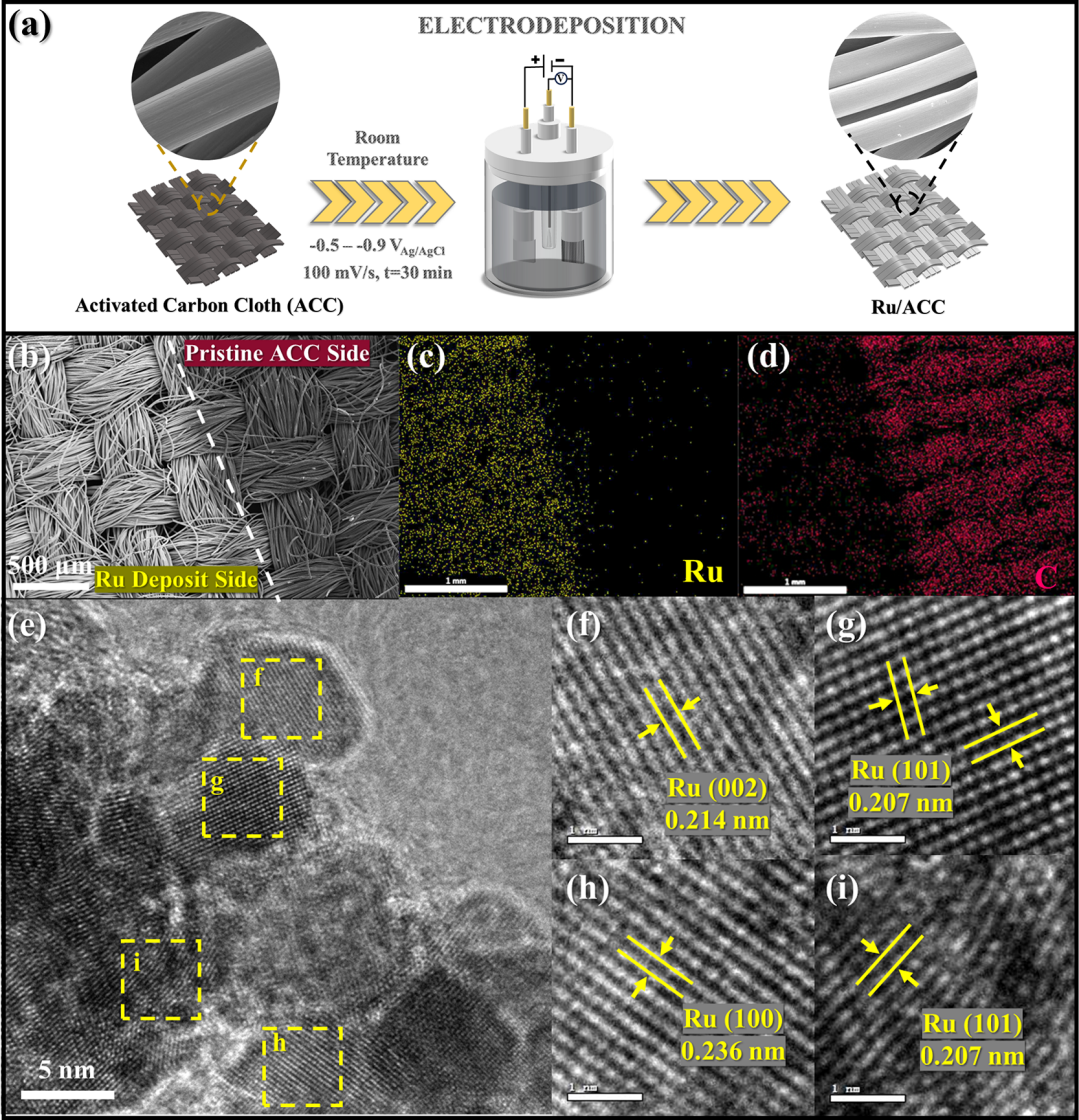
(a) Preparation of the Ru/ACC electrode. SEM images of the (b) boundary between electrodeposited and nonelectrodeposited ACC and (c,d) corresponding energy-dispersive X-ray (EDX) spectroscopy images of Ru and C. (e) HRTEM image of Ru/ACC, (f–i) enlarged HRTEM images and corresponding lattice spacings of marked areas in panel (e).

### 2.2 Electrocatalysis Procedures

All electrochemical reduction reactions were conducted in an H-type electrochemical cell (H-cell) divided by a Nafion 117 membrane (Figure S3). The working electrode (cathode) was the as-prepared Ru/ACC electrode with a 1 cm × 1 cm × 2 active area, paired with a Pt mesh anode with the same area. A synthetic landfill leachate was prepared using an existing procedure by dissolving tannic acid, which simulates the refractory organic matter content, and relevant inorganic matter in tap water.^31^

### 2.3 Product Analysis

Qualitative and partial quantitative analyzes of different refractory organics and their products were conducted through gas chromatography–mass spectrometry (GC–MS; Shimadzu 2010, Japan). The GC system was equipped with a Restek Rtx-5 column, 30 m × 0.32 mm with a 0.25 μm film thickness, 1.0 mL/min helium gas flow rate, and split ratio of 1:10. The injection temperature was set as 270 °C. The GC oven program began at 40 °C for 1 min, followed by heating at 15 °C/min to 260 °C. MS was conducted in electron ionization (EI) mode, capturing *m*/*z* from 28 to 400 with a sampling interval of 0.34 s. Chromatographic peaks were identified by referencing the mass spectra with the NIST library, and when necessary, by injecting an external standard. As the external standard, octane was used to identify compounds and quantify the peaks. Part of the aqueous soluble ECH product analysis was conducted using a high-performance liquid chromatography (HPLC; Waters, USA) device equipped with a photodiode array detector and reflective index detector, using an HPX-87H column (Aminex, Bio-Rad) at 60.0 °C. The mobile phase was 5 mM sulfuric acid in water flowing isocratically at 0.6 mL/min. The conversion rate (*Conv*), selectivity (*S*_*x*_), yield, carbon mass balance (CB) and Faraday efficiency (FE) were calculated using the following expressions:

$$Conv\;\left(\%\right)=\frac{{mol}_{reacted}}{{mol}_{Initial}}\times 100\%$$

$$S_{x}\;\left(\%\right)=\frac{{mol}_{x}}{{\Sigma mol}_{products}}\times 100\%$$

$$yield\;\left(\%\right)=\frac{{mol}_{x}}{{mol}_{reacted}}\times 100\%$$

$$CB\;\left(\%\right)=\frac{{\Sigma mol}_{products}+{mol}_{residual}}{{mol}_{initial}}\times 100\%$$

$$FE\;\left(\%\right)=\frac{n\times F\times \Delta N_{i}}{\int \int dIdt}\times 100\%$$

where mol_reacted_, mol_Initial_, and mol_residual_ denote the amounts of reacted, initial, and residual reactants, respectively. mol_*x*_ is the amount of the product of interest quantified in moles, and Σmol_products_ is the sum of all products, where *n* is the electron transfer number; *F* is the Faraday constant, 96,485 C mol^–1^; and Δ*N*_*i*_ (mol) is the number of moles of product *i*. The total charge was calculated from the integral of the cathodic current (*I*, A) with respect to the operation time (*t*, s).

### 2.4 Kinetic Luminescent Bacteria Test

The acute bioluminescence inhibition from sample constituents on a strain of *Aliivibrio fischeri* bacteria (formerly *Vibrio fischeri*) was carried out (Figure S4) according to EN ISO 21338 using a commercial kit BioTox LumoPlate. Five dilutions (100%, 50%, 25%, 12.5%, 6.25%) were used for each tested compound pair (i.e., before and after ECH) with three replicates per dilution to generate the dose–response curve. Phosphate buffer solution (PBS) with the same recipe of ECH reactions was used as the vehicle control (VC) while diluted as appropriate before being mixed with the bacteria suspension. The frozen bacteria were rehydrated before testing according to the manufacturer’s instructions. Immediately after mixing with samples, the 96-well plate was incubated for up to 45 min at 15 °C for effect determination. The luminescence was measured on a multimode microplate reader SpectraMax iD5 (Molecular Devices, CA, USA). The inhibition (%) were presented as the mean ± standard deviation (SD).

### 2.5 Biodegradation Denitrification Test

#### 2.5.1 Landfill SBR Sludge Preparation

The SBR active sludge was collected from the wastewater treatment facility of a local landfill known for reducing the NO_3_^–^ concentration in leachate to below 200 mg/L through biological denitrification. The SBR tank adopted a single-basin setup, in which nitrification and denitrification occur by alternating the aerobic and anoxic phases. The nitrifiers are dominantly aerobic and driven by oxidizing ammonia (NH_3_) or ammonium ions (NH_4_^+^) to nitrite (NO_2_^–^), followed by nitrite to nitrate. In denitrification, nitrate is sequentially reduced to nitrite, nitric oxide (NO), nitrous oxide (N_2_O) and resulting in the nitrogen (N_2_) formation.^32^

Nitrification

$${NH}_{4}^{+}+1.5O_{2}\to {NO}_{2}^{-}+2H^{+}+H_{2}O$$

$${NO}_{2}^{-}+0.5O_{2}\to {NO}_{3}^{-}$$

Denitrification

$${NO}_{3}^{-}\to {NO}_{2}^{-}\to NO\to N_{2}O\to N_{2}$$

This sludge was mainly used to treat landfill leachate characterized by high chemical oxygen demand (COD) that could overshadow the impact of tested organics. The COD content typically ranges from 3000 to 15,700 ppm.^33,34^ The raw sludge stocks were washed through seven runs of centrifugation (10,000 rpm, 2 min) at 20 ± 1 °C. KNO_3_ was added to the washed sludge to standardize the NO_3_^–^ concentration with relevant additives (Figure 3a). After being shaken evenly, the samples were incubated at 30 °C for 3 d during the model compound trials and 1 d for the ECH-treated electrolyte trial. The samples were then centrifuged and filtered with a 0.22 μm filter cartridge. Subsequently, the NO_3_^–^ content in the supernatant was assessed through ion chromatography (IC) analysis.

#### 2.5.2 Denitrification Rate Assessment

The washed sludge was transferred to centrifuge tubes, followed by dispensing of the testing solution, which was either the post-ECH electrolyte or a model compound solution with KNO_3_. After the designated period, the samples were centrifuged, and the NO_3_^–^, NO_2_^–^, and NH_4_^+^ content in the supernatant was analyzed through IC to assess the denitrification rate enhancement attributable to the presence of organic compounds within the solution. And total nitrogen (TN) was calculated as the sum of the measurements for ammonium, nitrite, and nitrate.^35^

$$Percentage\;change\;in\;{NO}_{3}^{-}\;\left(compared\;with\;no‐additive\;control,\%\right)$$

$$=\frac{C_{residual\;nitrate}-C_{residual\;nitrate\left(No‐additive\;control\right)}}{C_{residual\;nitrate\left(No‐additive\;control\right)}}\times 100\%$$

$$R=denitrification\;rate\;\left(mg\;L^{-1}\;h^{-1}\right)=\frac{C_{initial\;nitrate}-C_{residual\;nitrate}}{t}\times 100\%$$

$$denitrification\;rate\;enhancement\;\left(\%\right)=\frac{R_{after\;ECH}-R_{before\;ECH}}{R_{before\;ECH}}\times 100\%$$

where *C*_initial_ _nitrate_ and *C*_residual_ _nitrate_ denote the concentrations of the initial and residual nitrate, respectively; *t*(*h*) is the operation time; and *R*_before ECH_ and *R*_after ECH_ represent the denitrification rates of the samples before and after ECH, respectively.

A Dionex ICS-1100 system was used for the IC analysis. The IC system was equipped with an AS18 IonPac analytical column, AG18 guard column, and ASRS-300 suppressor for anion analysis. The samples were eluted with 20 mM KOH at 1 mL min^−1^. For cation analysis, the IC system was equipped with IonPac CS12A analysis column and IonPac CG12A guard column. Samples were eluted with 20 mM methanesulfonic acid (MSA) at 1 mL min^−1^.

### 2.6 Amplicon Sequencing and Quality Control

Genomic DNA was extracted using the PowerSoil Pro kit (Qiagen, USA), and the 16S rRNA gene V4 hypervariable region was amplified using 515*f*/806r primers.^36^ Each sample was assessed in triplicate. Sequencing was performed on an Illumina NovaSeq 6000 platform (Novogene, China) to generate 250-bp paired-end reads. A total of 2,547,651 raw reads were generated from all samples after merging paired-end reads using FLASH (v.1.2.11).^37^ Quality filtering of the raw reads was performed using fastp (v.0.23.1) with default settings,^38^ and chimeric sequences were removed using the UCHIME algorithm.^39^ On average, 64,648 ± 8911 high-quality reads were obtained per sample.

### 2.7 Bioinformatics

Amplicon sequence variants (ASVs) were generated and denoised using the DADA2 plugin^40^ in QIIME2 (v.2023.07).^41^ Taxonomic assignment of ASVs was performed using the SILVA 138 database (99% similarity).^42^ Alpha diversity metrics, including ASV richness and Pielou’s evenness, were calculated with the q2-diversity plugin in QIIME2 after rarefying samples to 34,600 reads. Differences in alpha diversity across different treatments and incubation days were analyzed using two-way ANOVA with Tukey’s posthoc test. Beta diversity analysis was performed using Bray–Curtis dissimilarity. Principal coordinate analysis was conducted with the “vegdist” function in the R package “vegan” (v.2.6–4), and PERMANOVA was applied using the “adonis2” function in “vegan” (999 permutations) to assess the effects of treatments and incubation days on bacterial composition. Nestedness (representing balanced variation) and turnover (representing unidirectional abundance gradients), key components of beta diversity, were calculated using the “bray.part” function in the R package “betapart” (v.1.6). A co-occurrence network was constructed based on strong (|ρ| > 0.6) and statistically significant (*p* < 0.001) correlations between ASVs (present in at least six samples) and nitrate concentrations. Correlations and *p*-values were computed with the “corAndPvalue” function in the R package “WGCNA”(v.1.72–5). Network construction was performed using the “graph_from_data_frame” function in the R package “igraph” (v.1.5.1), and node hub scores were calculated using the “hub_score” function. Network modularity was visualized using Gephi (v.0.10.1). Pairwise WRST was used to assess differences in diversity and taxonomy between groups. Statistical analyzes were performed in R (v.4.2.2) with significance set at *p* < 0.05.

## 3 Results and Discussion

### 3.1 Structural Characterization of Ru/ACC Electrodes

Metallic Ru, known for its ability to catalyze the ECH of aromatic compounds, was prepared on activated carbon cloth (ACC) via electrochemical deposition by cycling the working potential between −0.5 and −0.9 V_Ag/AgCl_ for 30 min. Scanning electron microscopy (SEM) images depicted the contrast between the surfaces before and after Ru electrodeposition, with lighter and darker areas representing the Ru deposit and pristine ACC surface, respectively (Figure 1b). The elemental mappings of Ru and C (Figure 1c,d) using energy-dispersive X-ray (EDX) spectroscopy further verified the elemental deposition. High-resolution transmission electron microscopy (HRTEM) (Figure 1e) investigations revealed the intimate contact between Ru and ACC. Clear lattice fringes with spacings of 0.207, 0.214, and 0.236 nm were observed, consistent with the (101), (002), and (100) planes of Ru, respectively. Similarly, X-ray diffraction (XRD) analysis (Figure S5) demonstrated the occurrence of peaks at 38.4°, 42.2°, 44.0°, and 58.3°, attributable to the (100), (002), (101), and (102) crystal planes of metallic Ru (JCPDS card no. 06-0663), respectively,^43^ in agreement with the HRTEM results. These results confirmed the successful deposition of Ru on the ACC surface.

### 3.2 Evaluation of ECH Performance

The ECH performance of the Ru/ACC electrode was evaluated using 20 mM 4-chlorophenol (4-CP) as the model contaminant in 0.5 M pH 7 phosphate buffer solution (PBS). During the ECH of 4-CP, a mixture of products, including phenol, cyclohexanone, and cyclohexanol, was detected, consistent with expectations for ECH treatment of chlorinated phenolics using platinum-group catalysts and pyrophoric Raney nickel.^44–47^ (Figure 2a) As the temperature increased, the conversion of 4-CP to cyclohexanol improved. However, 80 °C was selected as the upper bound because the electrolyte suffered significant loss from evaporation at higher temperatures, e.g., 90 °C. Moreover, the hydrogen evolution reaction (HER) was highly competitive at 90 °C, leading to suppressed ECH.^48^ Therefore, a temperature of 80 °C was used for subsequent investigations. Notably, setting the temperature at 80 °C may be advantageous when integrating the ECH with certain wastewater treatment plants. The landfill leachate in several regions contains a high nitrogen content, e.g., 2000–5000 mg L^–1^ in Hong Kong.^49^ Given that thermal stripping is typically conducted at 85–90 °C,^50^ the proposed ECH strategy exhibits excellent potential to be integrated with thermal stripping by leveraging the residual heat. The effects of different current densities (*j* = 10–70 mA cm^–2^) were examined at 80 °C. From 10 to 50 mA cm^–2^, 4-CP conversion to cyclohexanol was promoted. The conversion rate decreased at *j* = 70 mA cm^–2^ due to HER competition. Specifically, the conversion efficiency of 4-CP was reduced by 23.1% compared with that at *j* = 50 mA cm^–2^. Such reductions attributable to HER have been commonly observed in ECH studies.^51^

**Figure 2:**
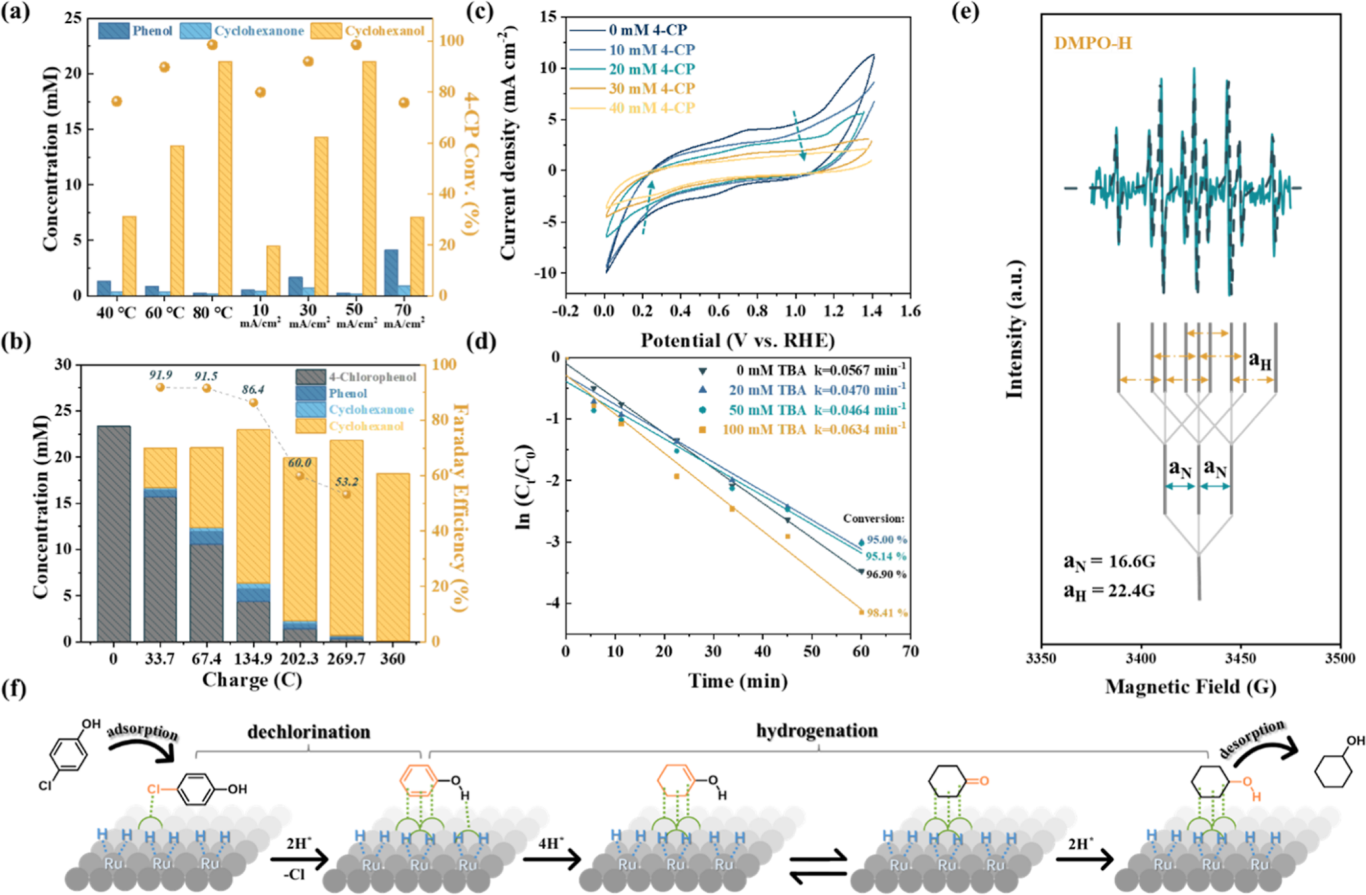
(a) Effect of temperature (°C) and current density (*j*) of 20 mM 4-CP in 0.5 M PBS (pH 7). The charge was fixed at 269.7 C, sufficient to reduce all 4-CP to cyclohexanol assuming perfect Faraday efficiency (FE). (b) Time-resolved electrolysis of 20 mM 4-CP at 80 °C and *j* = 50 mA cm^–2^. (c) Cyclic voltammetry (CV) scans of the Ru/ACC electrode in the presence of 4-CP at different concentrations. (d) Pseudo-first-order kinetic fitting curve, ln (*C*_*t*_/*C*_0_) = −*kt*, of ECH of 20 mM 4-CP in 0.5 M PBS (pH 7) with different concentrations of *tert*-butyl alcohol (TBA), a radical scavenger, at 80 °C and 50 mA cm^–2^. (e) Electron paramagnetic resonance (EPR) experimental (solid line) and simulation (dashed line) spectra, showing the hyperfine structure and Landé factor of the identified DMPO-H. (f) Proposed ECH reaction pathways of 4-CP on the Ru/ACC electrode.

Time-resolved electrolysis ECH of 4-CP was conducted at the optimized condition of 50 mA cm^–2^ at 80 °C (Figure 2b). The results confirmed that phenol, cyclohexanone, and cyclohexanol were the only products, with no benzene or cyclohexane formed during the reaction.^52^ According to the product distribution, phenol and cyclohexanone were the intermediates, maintaining a steady concentration throughout the reaction, and cyclohexanol, the terminal saturated product, grew at their expense. The FE, calculated based on the ECH products, was greatest in the initial stage with abundant reactants. Specifically, FE = 91.9% was achieved at 33.7 C, corresponding to approximately 5 min of electrolysis. The FE remained high (91.5%) at 67.4 C and gradually declined to 53.2% when 90.4% of 4-CP was converted to cyclohexanol. The low FE at the end of the reaction could be attributable to the insufficiency of reactants, which caused the ECH reaction to transition to the HER. Nonetheless, even at 90.4% conversion, the FE was maintained at 53.2%, comparable to other ECH reactions (FE: 49.5%–54.7%).^23,53,54^

The ECH electron transfer (ET) mechanism was investigated, specifically whether the reaction occurred through an inner- or outer-sphere ET mechanism. In contrast to inner-sphere ET, outer-sphere ET mechanisms tend to produce polymeric products due to radical-intermediate condensation. For example, during the ECH of furfural, outer-sphere-friendly materials such as lead, carbon felt, and 2H-phase MoS_2_ lead to the formation of hydrofuroin, a dimerized product,^55^ instead of the hydrogenated product, furfuryl alcohol. From the perspective of increasing the bioavailability of substrates, which are crucial for biological denitrification, hydrogenated products are preferred over polymerized products because the former decrease the degree of unsaturation, whereas the latter increase the molecular size and tend to reduce aqueous solubility and thus bioavailability.^56^ Inner-sphere ET mechanisms typically yield hydrogenated products because the reactants participate in direct surface–substrate interactions to obtain adsorbed hydrogen (H*). In contrast, outer-sphere ET allows the ET to occur at a distance from the electrode surface to produce a radical intermediate (R^•^), which then obtains H* from the electrode surface or electrolyte.

The presence of H* was observed using electron paramagnetic resonance (EPR) spectroscopy systems with 5,5-dimethyl-1-pyrroline *N*-oxide (DMPO) as the spin trap. A clear EPR signal for DMPO-H with nine characteristic peaks was observed for the Ru/ACC electrode, demonstrating that it can effectively generate H* (Figure 2e). The simulated spectrum with hyperfine splitting constants of α_N_ = 16.6 G and α_H_ = 22.4 G overlapped with the experimental EPR spectrum, confirming that the signal was generated from DMPO-H.^57^

The investigation into inner- vs outer-sphere ET mechanisms was conducted in a three-electrode system at a scan rate of 50 mV s^–1^. As shown in Figure 2c, the oxidation peaks between 1.0 and 1.4 *V*_RHE_ gradually declined in size and shifted to more positive values with the increasing concentration of 4-CP. This was attributable to the oxidation of H*, demonstrating the direct interaction between 4-CP and the Ru catalyst. In the negative scan, the reductive current was suppressed as the 4-CP concentration increased from 0 to 20 and 40 mM, with values of −7.1, −5.0, and −3.0 mA cm^–2^ at 0.1 *V*_RHE_, respectively. The marked decline in current suggests that 4-CP strongly adsorbed onto the H-generating sites and prevented the formation of H*, revealing that the Ru/ACC accomplished the ECH reaction through an inner-sphere ET mechanism. The surface interaction between H* and 4-CP was also verified via a series of control experiments with different concentrations of TBA acting as a H* quencher.^58^ In the presence of TBA (Figure 2d), the hydrogenation reaction on the Ru/ACC electrode was not affected. The 4-CP removal efficiency was 98.4% in the presence of 100 mM TBA (Figure S6), with only a slight fluctuation in the pseudo-first-order kinetic constant (from 0.0567 to 0.0634 min^–1^). These results confirmed that the ECH of 4-CP occurred on the electrode surface, with the electrolyte H* quencher not affecting the ECH efficiency. These outcomes indicate that inner-sphere ET was the ECH pathway on Ru/ACC.

Based on the detailed evaluation of ECH and insights from the literature,^23,29,59–61^ we propose a possible ECH reaction process of 4-CP (Figure 2f). Initially, 4-CP is adsorbed onto the Ru/ACC electrode surface. The ECH of 4-CP cleaves the C–Cl bond with one electron and one proton to yield an adsorbed chlorine atom (Cl*) and phenol. The Cl* is converted to Cl^–^ and desorbs from the surface. Subsequently, the aromatic ring of phenol is hydrogenated to yield cyclohexenol via two sets of two-electron and two-proton hydrogenations. The enol form of cyclohexenol undergoes tautomerization to cyclohexanone, which is hydrogenated to cyclohexanol. As the aromatic ring is saturated, the π bond interaction with the surface is diminished, leading to the eventual desorption of cyclohexanol. Meanwhile, incoming aromatic species take the place of the departed product to initiate a new ECH catalytic cycle. As the benzene ring becomes saturated, the strength of hydrogen-bond donor capacity is substantially decreased. This weakens the interaction between the OH and the electrode surface, allowing the final cyclohexanol product to leave the electrode surface rapidly. This vacancy is then occupied by a new 4-CP molecule, which binds more strongly. This process indicates that the Ru/ACC catalyst exhibits high dichlorination efficiency and superior hydrogenation capability.

### 3.3 ECH of Environmentally Relevant Aromatic Substrates and Ecotoxicity Comparison before and after ECH

After identifying the optimized reaction conditions and reaction mechanism for the ECH of aromatics, we attempted to broaden the scope of ECH conversion to various unsaturated compounds identifiable in landfill leachate, the paper and pulp industry, and phenolic wastewater.^27,62,63^ The ECH process demonstrated excellent (66.0%–100.0%) conversion rates with the hydrogenated product as the primary product (Table 1). The Ru/ACC electrodes effectively accomplished aromatic ring hydrogenation (Entries 1–2) and dehalogenation on the halogen-bearing compounds (Entries 7–12). Based on the product distribution, the ECH mechanism was the same as the 4-CP ECH pathway, with dehalogenation yielding phenol, followed by the formation of cyclohexanone and then cyclohexanol. Aldehydes readily underwent hydrogenation to produce a moderate yield of the corresponding primary alcohols (Entries 4–5). At the same time, cyclic olefin and nitro compounds were transformed to more-saturated hydrocarbons (Entry 3) and primary amines (Entry 6), respectively.

**Table 1:**
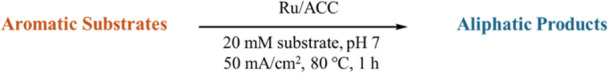

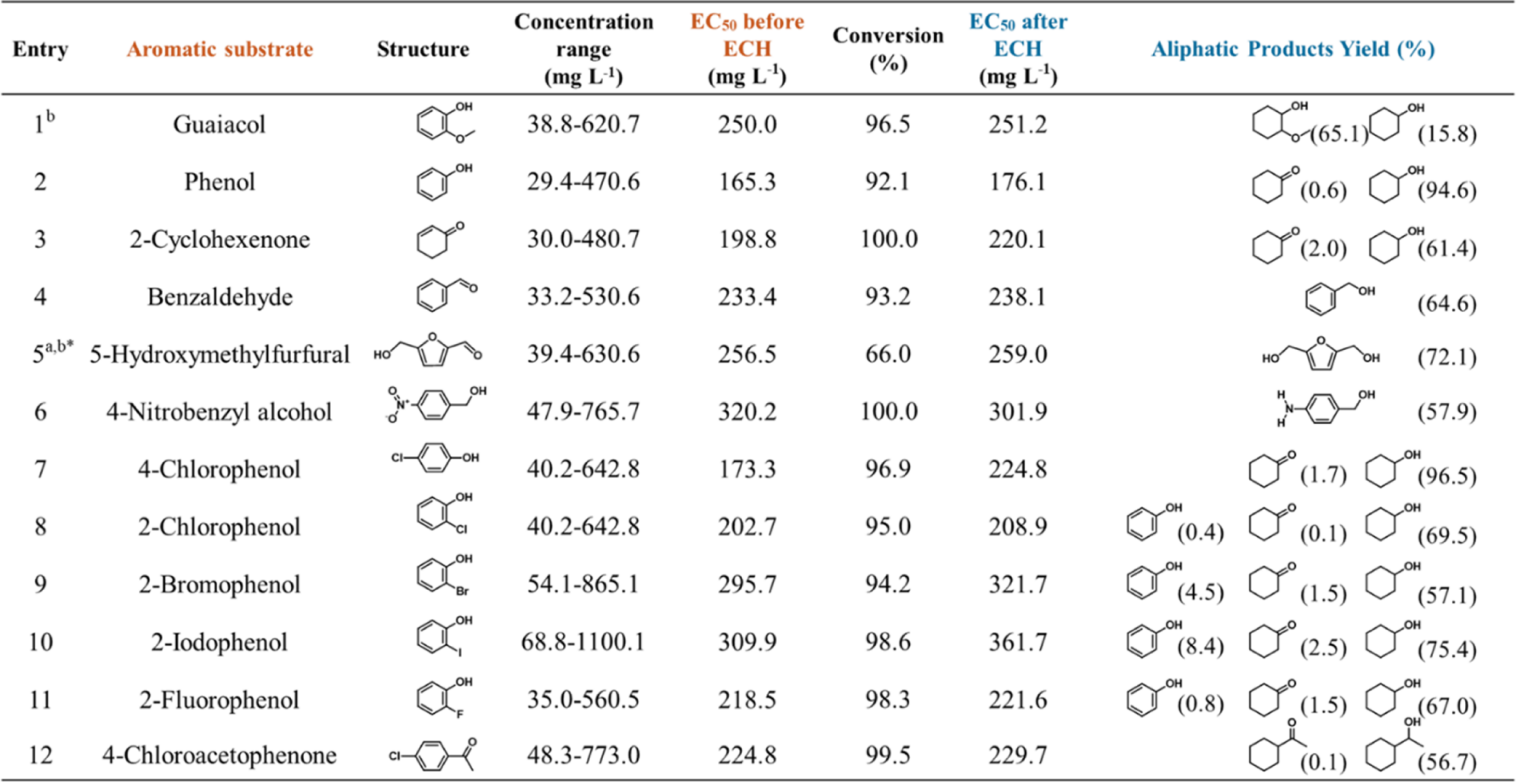
Electrocatalytic Hydrogenation of Various Contaminants Using the Ru/ACC Electrode at *j* = 50 mA cm^–2^ and 80 °C and Ecotoxicity Evaluated by Luminescent Bacteria Test (EC_50_, ISO 21338)^c^ ^,^ ^d^

a With ACN as a cosolvent in a ratio of 1:4 v/v % (ACN/buffer).

b 2 h reaction.

c Reaction conditions: Ru/ACC (geometric surface area of 1 cm × 1 cm) was used as the working electrode, and 20 mM contaminants were dissolved in pH 7 phosphate buffer.

d Unless specified otherwise, the substrates’ conversion and ECH product yield were determined and quantified with GC–MS using external standards. * was determined by HPLC due to their poor organic solvent extraction efficiency.

Considering the environmental impact, the ecotoxicity of the solution before and after ECH was further evaluated by the kinetic luminescent bacteria test (ISO 21338); concentration–response curves for EC_50_ measurements are also provided in Figure S4. Higher EC_50_ values indicate lower toxicity (Table 1). The results indicate that all solutions displayed a lower toxicity post-ECH treatment, as all their EC_50_ values increased, except for 4-nitrobenzyl alcohol, which displayed a minor (5.7%) drop in the EC_50_ value. These findings show that ECH treatment effectively reduces the toxicity of most aromatic compounds, demonstrating its potential for environment detoxification. These promising results indicate that the ECH technology described herein could be highly applicable to treating landfill leachate, in which the refractory organic content is abundant, with a soluble COD concentration of 6000–7000 mg L^–1^,^64^ including phenolic compounds, humic acids, and halogen-containing pollutants that are known to inhibit biological activity. Another potential application area is to treat paper and pulp industry wastewater discharged from the bleaching process stage, where chlorophenols and halogenated hydrocarbons and some lignin-derived aromatic compounds are present in abundance and typically display high toxicity to the exposed communities.^65^ By dechlorination and hydrogenation, our ECH protocol can greatly reduce the toxicity of discharged effluents while enhancing the bioavailable carbon source for the denitrification process.^66^

### 3.4 Enhanced Biological Denitrification

Heterotrophic denitrification, a process in which denitrifying bacteria utilize organic matter as electron donors and NO_3_^–^ as electron acceptors, is one of the most common methodologies for converting NO_3_^–^ to harmless N_2_.^67^ The bioavailability of organics is thus a critical factor in achieving efficient denitrification. The bioavailability of ECH-treated substrates as electron donors was assessed by comparing the changes in NO_3_^–^ concentration. As certain ECH reactions may result in multiple products, individual aromatic substrates and their expected ECH products were first assessed using model compounds. The sludge used in the test was obtained from a landfill SBR. The sludge was subjected to leachate denitrification and washed (see procedure and Figure 3a) before it was used in the denitrification biological test.

**Figure 3:**
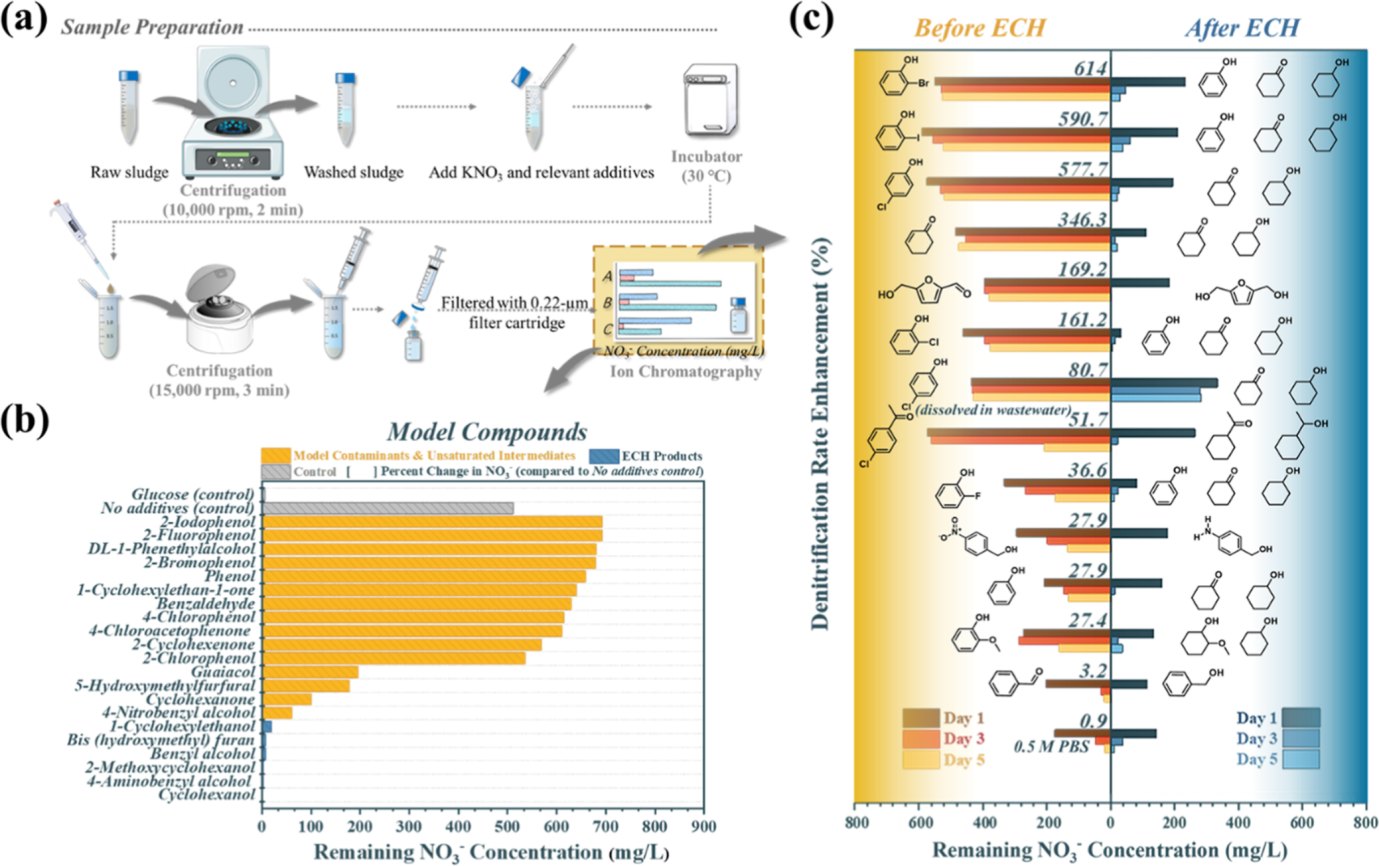
(a) Procedure of the biological denitrification test. Remaining NO_3_^–^ concentrations in (b) solutions with pure chemical additives after 3 day biodegradation at 30 °C. (c) Prereaction and neutralized postreaction solutions after 1 day, 3 day, and 5 day biodegradation.

After 3 day incubation, the NO_3_^–^ concentration of the glucose control dropped by 99.1%, decreasing from 664.2 to 5.5 mg L^–1^, signifying the high denitrification activity of the sludge (Figure 3b). The additive-free control showed only a 23.0% reduction in NO_3_^–^ concentration (final value of 511.5 mg L^–1^ NO_3_^–^), suggesting the presence of residual organics in the washed sludge. Meanwhile, the samples containing unsaturated compounds, such as phenol, 2-cyclohexenone, benzaldehyde, and halogen-containing substances, exhibited NO_3_^–^ concentrations greater than 511.5 mg L^–1^, indicating the inhibition of denitrification activity. In contrast, the expected ECH aliphatic products presented significantly lower NO_3_^–^ concentrations than the additive-free control (96.3–99.7% NO_3_^–^ reduction), and certain products, such as cyclohexanol and cyclohexylethanol, showed final NO_3_^–^ concentrations comparable to that of the glucose control (Figure 3b). These results confirm that the ECH products display higher bioavailability than their unsaturated forms.

The influence of ECH on the denitrification process for 12 compounds (Entries 1–12, Table 1) is illustrated in Figure 3c. Biological denitrification was assessed by comparing the NO_3_^–^ concentrations of the pre-ECH (left side) and neutralized post-ECH (right side) samples (Table S2) containing the model contaminant. The denitrification rate enhancement was calculated using data after a 5 day incubation period. As the incubation time increased from 1 to 5 days, the remaining NO_3_^–^ concentration in all samples decreased further. All hydrogenated contaminants exhibited varying amounts of remaining NO_3_^–^. Notably, 2-bromophenol, 2-iodophenol, and 4-CP exhibited remarkable decreases in the NO_3_^–^ content, indicating large denitrification-rate enhancements of 614.0%, 590.7%, and 577.7%, respectively. This result indicates that ECH treatment can also enable various dehalogenation reactions in addition to hydro-saturation, transforming toxic and resilient halogenated aromatics into biologically friendly aliphatics, which benefits denitrification. To simulate environmentally relevant conditions, the ECH treatment was also examined in real landfill leachate using 4-CP as a model reactant. The post-ECH leachate exhibited a significant denitrification-rate enhancement of 80.7%. The amount of organic product remaining after the 5 day denitrification was measured to confirm its consumability for denitrification. The amounts of all products, particularly cyclohexanol and 2-methoxycyclohexanol, showed significant decreases, indicating that they were consumed during the denitrification. Phenol and cyclohexylethan-1-one showed marginal decreases due to their unsaturated nature (Table S3). These results are consistent with their minor contributions to the denitrification process (Figure 3b). Interestingly, the amount of cyclohexanone increased in almost all the trials in which cyclohexanol was also a product. We speculated that the cyclohexanone was formed from oxidation of cyclohexanol, providing the electrons necessary for the denitrification step.

The nitrite and NH_3_ contents were also measured and are summarized in Figure S7. During denitrification, most samples showed small amounts of nitrite, an expected intermediate during denitrification. All samples showed only traces of NH_3_, except in Entry 5, where acetonitrile (ACN) was added as a cosolvent for 5-hydroxymethylfurfural to enhance its solubility (Table 1, Entry 5). A large amount of NH_3_ was formed due to the hydrolysis of ACN, which released NH_3_. Gas chromatography–mass spectrometry (GC–MS) analysis was performed for the pre- and post-ECH (Figure S8) electrolyte and revealed the production of acetamide, confirming the occurrence of ACN hydrolysis (for the mechanism, see Figure S9).^68^

### 3.5 Increase in Denitrifying Bacteria and Their Role in Bacterial Communities

The changes in bacterial communities in response to a model unsaturated contaminant (4-CP), its ECH-treated intermediate, phenol (PHE), and its saturated product, cyclohexanol (CYL), were investigated using 16S rRNA gene amplicon sequencing. During incubation, the NO_3_^–^ concentrations were monitored over 1 and 4 days across five treatments: blank condition (BLK), NO_3_^–^-only control (NO_3_), 4-CP, PHE, and CYL (Table S4). Only 4-CP and PHE retained significant amounts of NO_3_^–^, indicating inhibition of denitrification (Figure S10).

The bacterial community composition showed no significant variation between days 1 and 4 (Figure 4a), as supported by PERMANOVA (*R* ^2^ = 0.058, *p* = 0.127). The low turnover and nestedness values (<0.6) further indicated the dynamic stability of the bacterial community during this period (Figure 4b). In contrast, treatment type was the primary driver of variation in community composition (PERMANOVA, *R* ^2^ = 0.548, *p* = 0.001). Significant differences in alpha diversity metrics (richness and evenness) were observed among treatments (two- way ANOVA, *p* < 0.05; Figure 4c and Table S5), but not across incubation times (two-way ANOVA, *p* > 0.05). Specifically, bacterial diversity increased significantly in the NO_3_ treatment compared with BLK (Wilcoxon rank-sum test (WRST), *p* < 0.05). In contrast, diversity decreased significantly in the 4-CP and CYL treatments compared with NO_3_ (WRST, *p* < 0.05). Among treatments, CYL showed a slight but nonsignificant decrease in evenness (*p* > 0.05), with a significant difference observed only between CYL and 4-CP (WRST, *p* < 0.05). These findings suggest 4-CP, PHE, and CYL exert distinct effects on the bacterial community.

**Figure 4:**
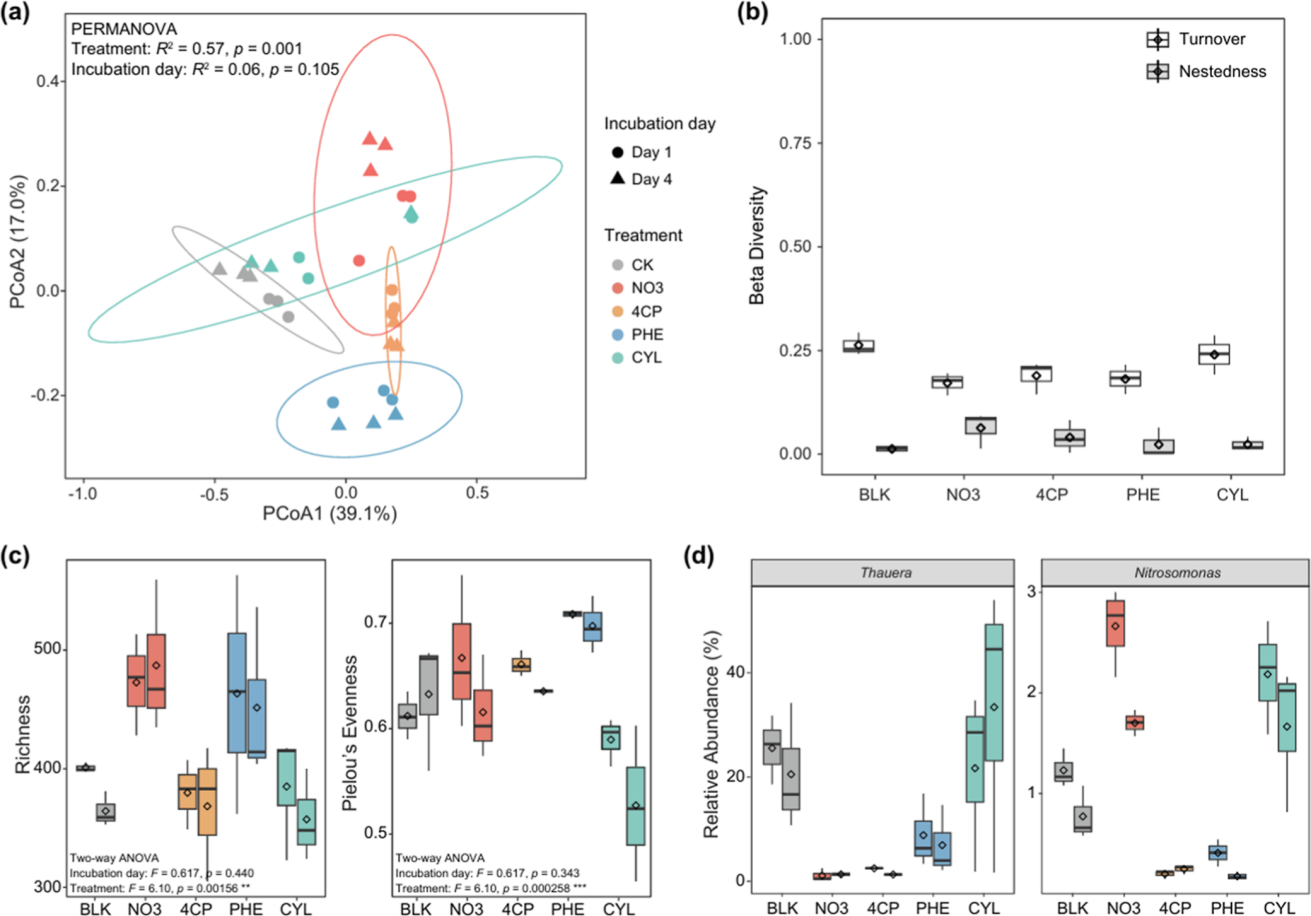
(a) Principal coordinate analysis of the Bray–Curtis dissimilarity matrix for bacterial communities across all samples. Ellipses represent 95% confidence intervals. (b) Beta diversity components (turnover and nestedness) of bacterial communities between days 1 and 4. (c) Alpha diversity metrics (richness and Pielou’s evenness) of bacterial communities. For each treatment—blank condition (BLK), nitrate-only control (NO_3_), 4-chlorophenol (4-CP), phenol (PHE), and cyclohexanol (CYL)—the left and right box bars represent days 1 and 4 of incubation, respectively. (d) Boxplots showing the relative abundances of a key denitrifier (*Thauera*) and a key nitrifier (*Nitrosomonas*) at the genus level across the five treatments. Boxplots display median, high, and low values, with the lower and upper edges of each box representing the first and third quartiles. The *x*-axis represents the treatment groups, and statistically significant differences between treatments are provided in Table S5.

The 10 most abundant bacterial genera across all samples were analyzed to identify key functional players under different treatments (Figure S11). Among these, members of the genus *Thauera* (ASV001 and ASV420), known for their association with denitrification,^69^ showed significantly higher relative abundance in the CYL treatment than in the NO_3_, 4-CP, and PHE treatments (WRST, *p* < 0.05; Figure 4d and Table S5). In the CYL treatment, the relative abundance of *Thauera* increased from an average of 21.7% ± 14.2% on day 1 to 33.8% ± 22.7% on day 4, potentially explaining the observed increase in community unevenness on day 4 (Figure 4c). This increase corresponds with the enhanced denitrification rate observed in the CYL treatment (Figure 3a,b), supporting previous findings that saturated hydrocarbons can enhance biological denitrification by specific bacteria.^70^ In contrast, *Thauera* showed no increase in the 4-CP and PHE treatments, consistent with the inhibited denitrification rates observed under these conditions (Figure 3a,b). Additionally, members of the genus *Nitrosomonas* (ASV019, ASV044, and ASV561), associated with nitrification,^71^ exhibited low relative abundance in the 4-CP and PHE treatments, suggesting potential inhibitory effects. However, their relative abundance was unaffected in the CYL treatment (Figure 4d).

To elucidate the role of NO_3_^–^-associated bacteria within the community under NO_3_^–^ addition, co-occurrence patterns between bacteria and NO_3_^–^ were analyzed across all samples. The resulting network was divided into seven major modules, with the top three modules accounting for 74.9% of the nodes (Figure 5a). NO_3_^–^ emerged as a central hub, ranking third in hub score and connecting to 55 amplicon sequence variants (ASVs) across the top four modules. Among these, two ASVs—ASV019 (affiliated with *Nitrosomonas*) and ASV043 (affiliated with *Nitrospira*)—exhibited particularly high hub scores, indicating strong interactions with other species in the network (Figure 5b). Similar to the *Nitrosomonas*-affiliated ASV019, the *Nitrospira*-affiliated ASV043, also associated with nitrification,^72^ showed low relative abundance under the 4-CP and PHE treatments but remained unaffected in the CYL treatment (Figure 5c). These findings suggest that the CYL treatment effectively alleviates the inhibitory effects of 4-CP and PHE on the growth of nitrifying and denitrifying bacteria.

**Figure 5:**
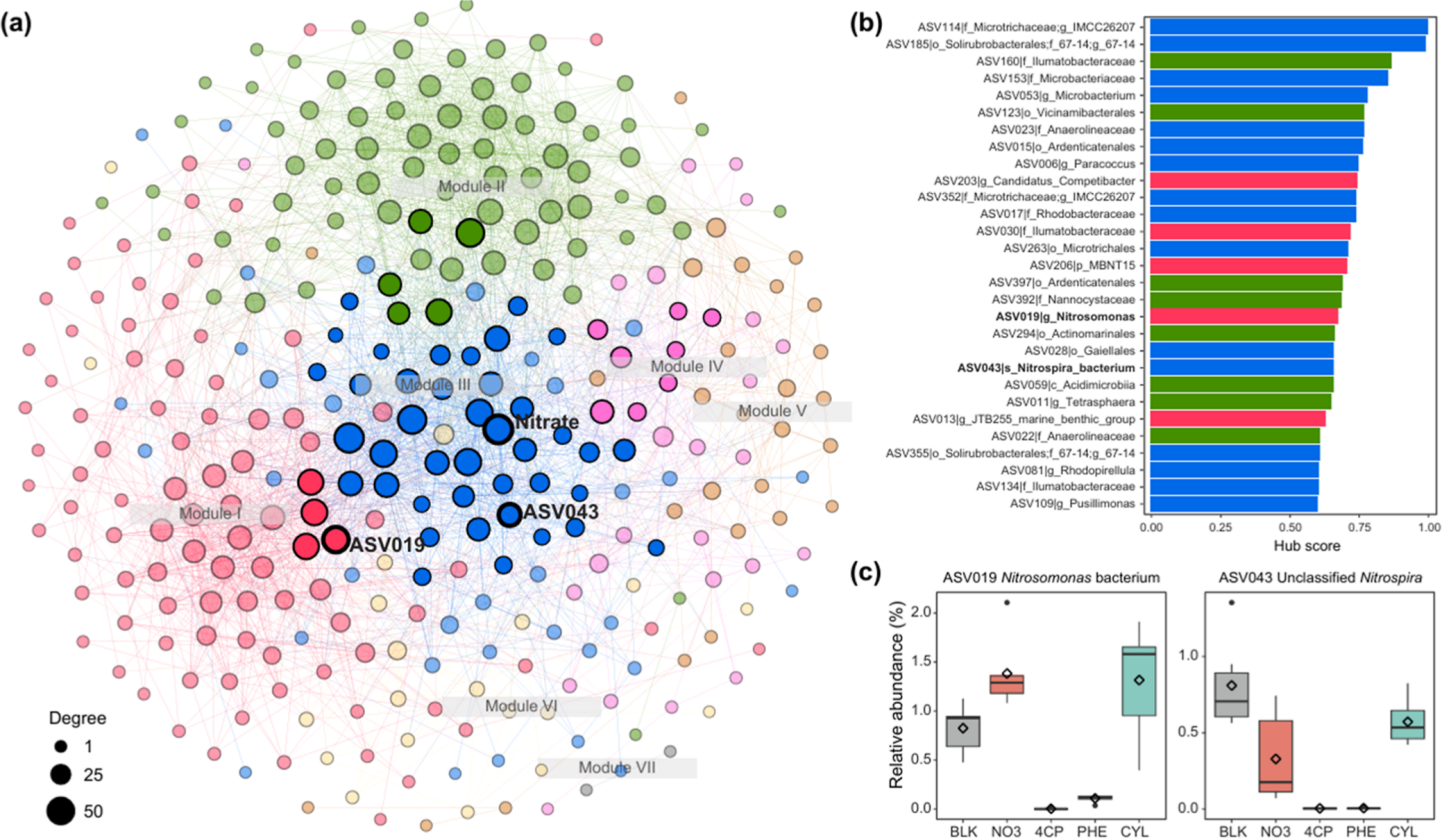
(a) Co-occurrence network analysis showing correlations between ASVs and nitrate. Only strong (Spearman’s |ρ| > 0.6) and statistically significant (*p* < 0.001) correlations are included. Node size represents the number of connections (degree). (b) Nodes (ASVs) with high hub scores (>0.6) are shown, with bars colored by modules and the two nitrate-associated ASVs highlighted in bold. (c) Relative abundances of the two nitrate-associated ASVs across the five treatments.

### 3.6 Application Potential of ECH in Wastewater Treatment

To explore the adaptability of the designed ECH treatment in real wastewater scenarios, we first investigated the effects of pH, organic compounds, and coexisting ion compositions.^23,66^ As shown in Figure 6a–e, our ECH treatment demonstrated excellent adaptability across a wide pH range of 3–11. Notably, our system performed even better under alkaline conditions, which are typically unfavorable to AOP-type reactions. Within 1 h, 99.0% of 4-CP was successfully transformed at pH 11, and under acidic conditions (pH 3), 86.8% 4-CP conversion was achieved. The minor performance decline in acidic conditions is attributable to the competing HER. We also investigated the effect of different supporting electrolytes. Electrolytes with buffering effects (PBS and (NH_4_)_2_SO_4_) showed a similar trend, with the conversion rate increasing steadily. However, for the nonbuffering electrolytes, such as KCl and K_2_SO_4_, the conversion rate plateaued quickly, with over 95% of 4-CP successfully transformed within just 20 min. It is possible that the lack of a buffering effect allowed the pH to increase quickly, which enhanced the ECH process. Meanwhile, the results also demonstrate that the coexistence of SO_4_^2–^, Cl^–^, PO_4_^3–^, and NH_4_^+^ has no observable influence on our system. The potential effect of organic compounds was also investigated. There was no significant change in performance in the presence of sodium alginate or sodium dodecyl sulfate. However, humic acid slowed the reaction rate, likely due to its strong adsorbing property,^73^ which enabled it to occupy active reaction sites and limit the 4-CP conversion process.

**Figure 6:**
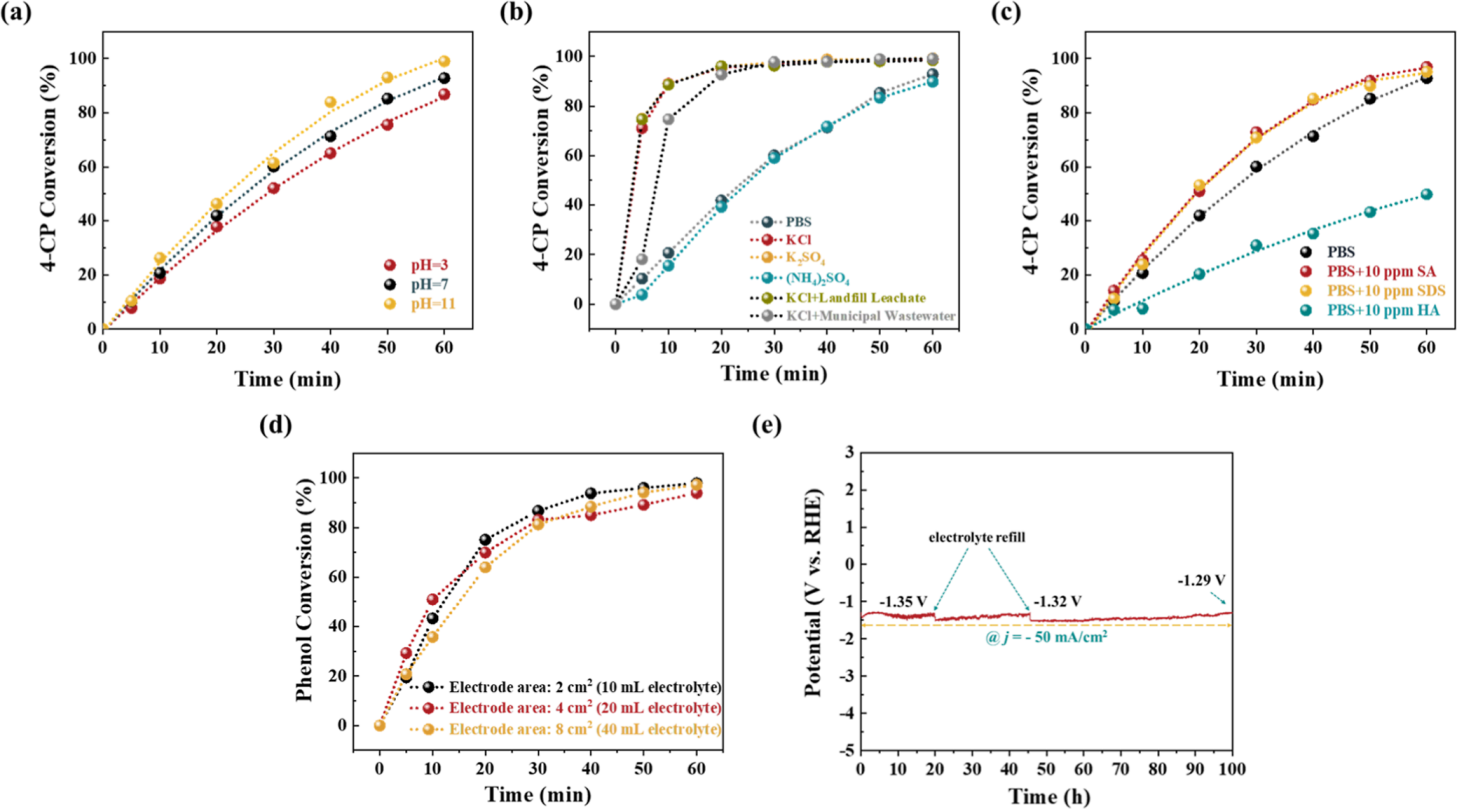
Effect of different solution conditions on the ECH performance of the Ru/ACC electrode: (a) pH (The pH of the catholyte was adjusted by adding aliquots of 1.0 M H_3_PO_4_ or NaOH. The anolyte was 0.5 M PBS (pH = 7)), (b) supporting electrolyte (0.5 M), and (c) coexisting substances (10 ppm). (d) Electrode dimension and treatment capacity. (e) Chronopotentiometry plot of Ru/ACC for 100 h at a current density of 50 mA cm^–2^ in landfill leachate.

To further simulate the complex background of real leachate, we dissolved 4-CP separately into real leachate and pretreated municipal wastewater, and added the necessary salt (Figure 6b) for conductivity. The 4-CP conversion remained greater than 95% within 30 min. These results further indicate that our ECH system has high applicability to the treatment of real wastewater.

The stability of Ru/ACC was assessed using cyclic voltammetry (CV) analysis (Figure S12). The results indicated that the Ru/ACC maintained its polarization curve even after 1000 CV scan cycles. In addition, a 100 h chronopotentiometry analysis was conducted at 50 mA cm^–2^ in a real landfill leachate (composition shown in Table S6). (Figure 6e) A slight, continual rise in potential occurred, likely due to the diminished concentration of oxidizable organics over time. This hypothesis was confirmed when the potential returned to its initial value after the electrolyte was refilled. After 100 h electrolysis, inductively coupled plasma optical emission spectrometry (Text S2) detected no Ru content in the electrolyte (Table S7). These results demonstrate the good activity and excellent stability of Ru/ACC in streams with complex compositions, indicating its significant potential for actual wastewater pretreatment application.

The operational feasibility and scaling-up potential were investigated. The electrode dimensions and electrolyte volume were proportionally increased from 2 cm^2^/10 mL to 4 cm^2^/20 mL and 8 cm^2^/40 mL while maintaining constant current density (50 mA cm^–2^) and phenol concentration (20 mM). As shown in Figure 6d, all scaled systems exhibited comparable phenol conversion profiles, achieving over 90% transformation efficiency. This linear correlation between electrode area and treatment capacity implies promising scalability potential. Meanwhile, critical analysis of substrate concentration-to-current density ratios (Figure S13) revealed that decreasing this ratio promoted competitive HER, causing proportional reductions in hydrogenation kinetics. These findings establish fundamental guidelines for industrial implementation while highlighting the necessity of optimizing substrate concentration-to-current density relationships for suppressing the competing HER.

**Table 2:** Cost-Effectiveness Comparison of Different Techniques for the Treatment of Phenolic Wastewater

technique	phenol concentration (mM)	current density (mA cm^–2^)	average potential (V)	kinetic constant (min^–1^)	*T* (h)	energy consumption (kWh)	energy consumption (kWh m^–3^)	cost^c^ ($ m^–3^)	pH adjustment	hazardous waste	pollutant valorization
ECH^a^	10	25	4.37	0.0664	0.67	16.62	7.32	∼0.56	No	No	Yes
	10	50	8.74	0.0337	1	93.54	43.70	∼3.34			
	20	50	9.44	0.0658	0.67	35.83	31.62	∼2.42			
EO^b^	0.52	20			1	51–269			No	No	No
O3^b^	20							∼5.38	No	No	No
Fenton^b^	20							∼3.92	Yes	Yes	No

a The data were based on the experimental reaction, which was conducted in an H-cell reactor with 20 mL of solution.

b The data on O_3_ and Fenton reactions were derived from previous studies.^66,74,75^

c The costs of ECH were calculated based on an electricity price of 0.0765 $ kWh^–1^.^74^

Cost is a major consideration in developing novel technologies, as it influences processing expenses and commercial viability. To ensure a positive impact on the wastewater denitrification process, we assessed the cost-effectiveness of the ECH technology using an H-cell reactor (Table 2). The reaction kinetics demonstrated comparable treatment rates between systems operating at 20 mM phenol/50 mA cm^–2^ (0.0664 min^–1^) and 10 mM phenol/25 mA cm^–2^ (0.0658 min^–1^), with energy consumption scaling linearly with applied current density. Additionally, increasing the phenol concentration from 10 to 20 mM reduced the energy consumption by 61.7% (93.54 to 35.83 kWh kg^–1^ phenol). Despite the elevated average cell potential induced by the Nafion membrane, the energy consumption remains relatively low compared with most electrooxidation processes, which typically consume between 51 and 269 kWh kg^–1^ phenol.^75^ This energy consumption can vary based on factors such as the initial reactant concentration, supporting electrolyte type and amount, and the applied current density or potential.

For the removal of 20 mM phenol, the cost is approximately 2.42 $ m^–3^, which is comparable to the costs of conventional AOPs such as ozonation (5.38 $ m^–3^) and Fenton oxidation (3.92 $ m^–3^). In our system, phenol is hydrogenated to cyclohexanol, transforming waste pollutants into valuable products or carbon sources for denitrifying bacteria. Additionally, the aromatic-containing solution post-treatment can significantly enhance denitrification. Assuming a daily treatment of 100,000 gallons of wastewater with an influent NO_3_–N concentration of 20 mg L^–1^, the system can meet the environmental target of reducing the NO_3_–N concentration in discharged water to below 10 mg L^–1^ within a day (Tables S8 and S9). Based on the 80.7% denitrification rate enhancement observed with 4-CP in wastewater (Figure 3c), the pre-ECH system can save 0.45 days per 1 day treatment, saving approximately $23.12–$129.67 day^–1^ (Table S10).

It may be argued that methanol, a widely used external carbon source, would cost around $11.78–$14.13 day^–1^ to achieve a similar denitrification enhancement, making it cheaper than our ECH system ($24.23–$32.23 day^–1^). However, ECH treatment for transforming refractory pollutants into bioavailable stocks converts pollutants into valuable substances, while methanol poses safety concerns such as flammability and explosion hazards. To further enhance the cost-effectiveness of the pre-ECH system, we will explore the application of the anode side in our H-cell system, develop corresponding batch cell designs and trials, and integrate renewable electricity inputs, making it a green and effective wastewater treatment solution.

## 4 Implications

This study introduces a proof-of-concept ECH approach that valorizes aqueous bioinhibiting aromatics to enhance biodegradation for denitrification. Instead of viewing pollutants as chemical hazards and mineralizing them through an energy- and chemical-intensive protocol, this study ushers in a new paradigm based on ECH to transform pollutants into desirable substances. The proposed electrocatalyst operates through an inner-sphere ET mechanism that favors the formation of saturated products. The ECH exhibits high activity over a wide range of pH and extended electrolysis durations, enabling 66.0%–100.0% conversion with a 56.8%–98.2% expected product yield over an array of biodenitrification-inhibiting unsaturated, aromatic, and halogenated aromatic compounds. The saturated aliphatic compounds resulting from ECH promote the growth of denitrifiers (e.g., *Thauera*) and biological denitrification (3.2%–614.0%). Operating under atmospheric conditions below the boiling temperature of water, the proposed approach does not require sophisticated equipment, while an optimal temperature of 80 °C offers the potential to leverage residual heat from thermal NH_3_ stripping treatments. Based on the preliminary cost-effectiveness results, this innovative approach not only significantly reduces the time and economic costs associated with conventional denitrification methods but also showcases considerable potential for practical application in wastewater treatment.

## Supplementary Material



## Notes

Summary table of the main acronyms, pH monitoring of electrolytes, aliphatic products transformation after denitrification process, conditions for the BLK, NO_3_, 4-CP, PHE, and CYL treatment, pairwise comparisons using pairwise Wilcoxon rank sum test, wastewater information, ICP-OES method and data, electrode characterizations, electrochemical tests, concentration–response curves for EC_50_ measurements, nitrogen (nitrate, nitrite, ammonia nitrogen, and total nitrogen) information after denitrification incubation, the average relative abundance of the top 10 genera in bacterial communities, cost-effectiveness analysis and other detailed information (PDF)

## References

[1] Sawyerr N., Trois C., Oyebode O., Bwapwa J. K. (2021). Denitrification
of leachate using composted domestic waste at different levels of
stability: A batch test investigation. Sci.
Afr..

[2] Mishra S., Singh V., Cheng L., Hussain A., Ormeci B. (2022). Nitrogen removal
from wastewater: A comprehensive review of biological nitrogen removal
processes, critical operation parameters and bioreactor design. J. Environ. Chem. Eng..

[3] Zhou Y., Zhu Y., Zhu J., Li C., Chen G. (2023). A Comprehensive Review
on Wastewater Nitrogen Removal and Its Recovery Processes. Int. J. Environ. Res. Public Health.

[4] Pang Y., Wang J. (2021). Various electron donors for biological nitrate removal: A review. Sci. Total Environ..

[5] Dietrich M. J., Randall T. L., Canney P. J. (1985). Wet air
oxidation of hazardous organics
in wastewater. Environ. Prog..

[6] Priac A., Morin-Crini N., Druart C., Gavoille S., Bradu C., Lagarrigue C., Torri G., Winterton P., Crini G. (2017). Alkylphenol and alkylphenol
polyethoxylates in water and wastewater:
A review of options for their elimination. Arabian
J. Chem..

[7] Wang Y.-f., Wu Y., Pi N., Tam N. F.-y. (2014). Investigation of microbial community
structure in constructed mangrove microcosms receiving wastewater-borne
polycyclic aromatic hydrocarbons (PAHs) and polybrominated diphenyl
ethers (PBDEs). Environ. Pollut..

[8] Ramos C., Fernández I., Suárez-Ojeda M.
E., Carrera J. (2015). Inhibition
of the anammox activity by aromatic compounds. Chem. Eng. J..

[9] Liang S., Wang J., Shen Z., Yan W., Nengzi L., Feng C., Lei X., Yu L., Hu J. (2024). Efficiently
removing dissolved organic pollutants in landfill leachate concentrate
using dual-anode Fe2+/HClO system: The significance of insolubilization
based on oxidative coupling of organics. Sep.
Purif. Technol..

[10] Slack R. J., Gronow J. R., Voulvoulis N. (2005). Household
hazardous waste in municipal
landfills: contaminants in leachate. Sci. Total
Environ..

[11] Mrowiec, B. ; Suschka, J. Presence and Effects of Aromatic Hydrocarbons on Sewage Treatment Efficiency; University of Massachusetts Amherst, 2010; .

[12] Nzila A. (2018). Current Status
of the Degradation of Aliphatic and Aromatic Petroleum Hydrocarbons
by Thermophilic Microbes and Future Perspectives. Int. J. Environ. Res. Public Health.

[13] Kamal A., Makhatova A., Yergali B., Baidullayeva A., Satayeva A., Kim J., Inglezakis V. J., Poulopoulos S. G., Arkhangelsky E. (2022). Biological Treatment, Advanced Oxidation
and Membrane Separation for Landfill Leachate Treatment: A Review. Sustainability.

[14] Rayaroth M. P., Aravindakumar C. T., Shah N. S., Boczkaj G. (2022). Advanced oxidation
processes (AOPs) based wastewater treatment - unexpected nitration
side reactions - a serious environmental issue: A review. Chem. Eng. J..

[15] Ren M., Sun S., Wu Y., Shi Y., Wang Z. J., Cao H., Xie Y. (2022). The structure-activity
relationship of aromatic compounds in advanced
oxidation processes:a review. Chemosphere.

[16] Glaze W. H., Kang J.-W., Chapin D. H. (1987). The Chemistry
of Water Treatment
Processes Involving Ozone, Hydrogen Peroxide and Ultraviolet Radiation. Ozone: Sci. Eng..

[17] Priyadarshini M., Das I., Ghangrekar M. M., Blaney L. (2022). Advanced oxidation
processes: Performance, advantages, and scale-up of emerging technologies. J. Environ. Manage..

[18] Pandis P. K., Kalogirou C., Kanellou E., Vaitsis C., Savvidou M. G., Sourkouni G., Zorpas A. A., Argirusis C. (2022). Key Points
of Advanced Oxidation Processes (AOPs) for Wastewater, Organic Pollutants
and Pharmaceutical Waste Treatment: A Mini Review. ChemEngineering.

[19] Tufail A., Price W. E., Hai F. I. (2020). A critical
review on advanced oxidation
processes for the removal of trace organic contaminants: A voyage
from individual to integrated processes. Chemosphere.

[20] Belhadj
Tahar N., Abdelhedi R., Savall A. (2009). Electrochemical polymerisation
of phenol in aqueous solutionon a Ta/PbO2 anode. J. Appl. Electrochem..

[21] Wu X., Wang Z., Zhang D., Qin Y., Wang M., Han Y., Zhan T., Yang B., Li S., Lai J., Wang L. (2021). Solvent-free microwave synthesis of ultra-small Ru-Mo2C@CNT with
strong metal-support interaction for industrial hydrogen evolution. Nat. Commun..

[22] Hossain M. A., Saelee T., Tulaphol S., Rahaman M. S., Phung T. K., Maihom T., Praserthdam P., Praserthdam S., Yelle D. J., Sathitsuksanoh N. (2022). Catalytic
Hydrogenolysis of Lignin
into Phenolics by Internal Hydrogen over Ru Catalyst. ChemCatChem.

[23] Ni N., Gu Z., Kang Y., Zhu D., Mao J., Wu K., Hu C. (2023). Electrocatalytic deep hydrogenation of 4-chlorophenol into cyclohexanol
on microchannel-enhanced Ru/TiO2 for wastewater detoxification and
simultaneous resource recovery. J. Environ.
Chem. Eng..

[24] Chaudhari C., Sato K., Miyahara S. i., Yamamoto T., Toriyama T., Matsumura S., Kusuda K., Kitagawa H., Nagaoka K. (2022). The Effect
of Ru Precursor and Support on the Hydrogenation of Aromatic Aldehydes/Ketones
to Alcohols. ChemCatChem.

[25] Sun R., Guo H.-Y., Ma S.-S., Wang Y.-F., Yu Z.-K., Xu B.-H. (2022). Ru­(dppbsa)-catalyzed hydrodeoxygenation and reductive etherification
of ketones and aldehydes. Org. Chem. Front..

[26] Fulignati S., Antonetti C., Wilbers E., Licursi D., Heeres H. J., Raspolli
Galletti A. M. (2021). Tunable HMF hydrogenation to furan diols in a flow
reactor using Ru/C as catalyst. J. Ind. Eng.
Chem..

[27] Mohd A. (2022). Presence of
phenol in wastewater effluent and its removal: an overview. Int. J. Environ. Anal. Chem..

[28] Kordek K., Jiang L., Fan K., Zhu Z., Xu L., Al-Mamun M., Dou Y., Chen S., Liu P., Yin H., Rutkowski P., Zhao H. (2018). Two-Step Activated Carbon Cloth with
Oxygen-Rich Functional Groups as a High-Performance Additive-Free
Air Electrode for Flexible Zinc–Air Batteries. Adv. Energy Mater..

[29] Wang Y., Yao J., Li H., Su D., Antonietti M. (2011). Highly selective
hydrogenation of phenol and derivatives over a Pd@carbon nitride catalyst
in aqueous media. J. Am. Chem. Soc..

[30] Kutyła D., Kołczyk K., Kowalik R., Żabiński P. (2016). Electrochemical
Deposition of Ruthenium and Cobalt-Ruthenium Alloys From Acidic Chloride
Ions Containing Baths. Arch. Metall. Mater..

[31] Ren Y., Ferraz F., Kang A. J., Yuan Q. (2017). Treatment of old landfill
leachate with high ammonium content using aerobic granular sludge. J. Biol. Eng..

[32] Li Y., Wang S., Li H., Fang Y., Yang L., Su F. (2020). Contribution of co-denitrification
to nitrous oxide production from
subsurface wastewater infiltration system. Ecol.
Eng..

[33] Fang H. H. P., Lau I. W. C., Wang P. (2005). Anaerobic
treatment of Hong Kong
leachate followed by chemical oxidation. Water
Sci. Technol..

[34] Lo I. M. C. (1996). Characteristics
and treatment of leachates from domestic landfills. Environ. Int..

[35] Li C., Wu S., Dong R. (2015). Dynamics of organic matter, nitrogen
and phosphorus
removal and their interactions in a tidal operated constructed wetland. J. Environ. Manage..

[36] Caporaso J. G., Lauber C. L., Walters W. A., Berg-Lyons D., Lozupone C. A., Turnbaugh P. J., Fierer N., Knight R. (2011). Global patterns
of 16S rRNA diversity at a depth of millions of sequences per sample. Proc. Natl. Acad. Sci. U.S.A..

[37] Magoč T., Salzberg S. L. (2011). FLASH: fast length
adjustment of short reads to improve
genome assemblies. Bioinformatics.

[38] Bokulich N. A., Subramanian S., Faith J. J., Gevers D., Gordon J. I., Knight R., Mills D. A., Caporaso J. G. (2013). Quality-filtering
vastly improves diversity estimates from Illumina amplicon sequencing. Nat. Methods.

[39] Edgar R. C., Haas B. J., Clemente J. C., Quince C., Knight R. (2011). UCHIME improves
sensitivity and speed of chimera detection. Bioinformatics.

[40] Callahan B. J., McMurdie P. J., Rosen M. J., Han A. W., Johnson A. J. A., Holmes S. P. (2016). DADA2: High-resolution sample inference
from Illumina
amplicon data. Nat. Methods.

[41] Bolyen E., Rideout J. R., Dillon M. R., Bokulich N. A., Abnet C. C., Al-Ghalith G. A., Alexander H., Alm E. J., Arumugam M., Asnicar F., Bai Y., Bisanz J. E., Bittinger K., Brejnrod A., Brislawn C. J., Brown C. T., Callahan B. J., Caraballo-Rodríguez A. M., Chase J., Cope E. K., Da Silva R., Diener C., Dorrestein P. C., Douglas G. M., Durall D. M., Duvallet C., Edwardson C. F., Ernst M., Estaki M., Fouquier J., Gauglitz J. M., Gibbons S. M., Gibson D. L., Gonzalez A., Gorlick K., Guo J., Hillmann B., Holmes S., Holste H., Huttenhower C., Huttley G. A., Janssen S., Jarmusch A. K., Jiang L., Kaehler B. D., Kang K. B., Keefe C. R., Keim P., Kelley S. T., Knights D., Koester I., Kosciolek T., Kreps J., Langille M. G. I., Lee J., Ley R., Liu Y.-X., Loftfield E., Lozupone C., Maher M., Marotz C., Martin B. D., McDonald D., McIver L. J., Melnik A. V., Metcalf J. L., Morgan S. C., Morton J. T., Naimey A. T., Navas-Molina J. A., Nothias L. F., Orchanian S. B., Pearson T., Peoples S. L., Petras D., Preuss M. L., Pruesse E., Rasmussen L. B., Rivers A., Robeson M. S., Rosenthal P., Segata N., Shaffer M., Shiffer A., Sinha R., Song S. J., Spear J. R., Swafford A. D., Thompson L. R., Torres P. J., Trinh P., Tripathi A., Turnbaugh P. J., Ul-Hasan S., van der Hooft J. J. J., Vargas F., Vázquez-Baeza Y., Vogtmann E., von Hippel M., Walters W., Wan Y., Wang M., Warren J., Weber K. C., Williamson C. H. D., Willis A. D., Xu Z. Z., Zaneveld J. R., Zhang Y., Zhu Q., Knight R., Caporaso J. G. (2019). Reproducible, interactive, scalable
and extensible microbiome data science using QIIME 2. Nat. Biotechnol..

[42] Quast C., Pruesse E., Yilmaz P., Gerken J., Schweer T., Yarza P., Peplies J., Glöckner F. O. (2012). The SILVA
ribosomal RNA gene database project: improved data processing and
web-based tools. Nucleic Acids Res..

[43] Li Y., Zhang L. A., Qin Y., Chu F., Kong Y., Tao Y., Li Y., Bu Y., Ding D., Liu M. (2018). Crystallinity
Dependence of Ruthenium Nanocatalyst toward Hydrogen Evolution Reaction. ACS Catal..

[44] Li Y., Liu Y., Zhang X., Tian K., Tan D., Song X., Wang P., Jiang Q., Lu J. (2022). Electrochemical
Reduction
and Oxidation of Chlorinated Aromatic Compounds Enhanced by the Fe-ZSM-5
Catalyst: Kinetics and Mechanisms. ACS Omega.

[45] Mao R., Huang C., Zhao X., Ma M., Qu J. (2019). Dechlorination
of triclosan by enhanced atomic hydrogen-mediated electrochemical
reduction: Kinetics, mechanism, and toxicity assessment. Appl. Catal. B.

[46] Wu H., Chen H., Yu C., Qu R., Shi M., Lou Z., Zhu J., Yu J., Xu Y. (2023). New pattern of Pd-Catalyzed
electrochemical hydrodechlorination in conversion of chlorinated aromatic
pollutants to Value-Added chemicals. Chem. Eng.
J..

[47] Yang B., Yu G., Liu X. (2006). Electrochemical
hydrodechlorination of 4-chlorobiphenyl
in aqueous solution with the optimization of palladium-loaded cathode
materials. Electrochim. Acta.

[48] Roessler A., Dossenbach O., Rys P. (2003). Electrocatalytic Hydrogenation of
Indigo Process Optimization and Scale-Up in a Flow Cell. J. Electrochem. Soc..

[49] Li X. Z., Zhao Q. L. (2003). Recovery of ammonium-nitrogen from landfill leachate
as a multi-nutrient fertilizer. Ecol. Eng..

[50] Leverenz H., Adams R., Hazard J., Tchobanoglous G. (2021). Continuous
Thermal Stripping Process for Ammonium Removal from Digestate and
Centrate. Sustainability.

[51] Bai L., Wang Y., Han Z., Bai J., Leng K., Zheng L., Qu Y., Wu Y. (2023). Efficient industrial-current-density
acetylene to polymer-grade ethylene via hydrogen-localization transfer
over fluorine-modified copper. Nat. Commun..

[52] Zhao B., Guo Q., Fu Y. (2014). Electrocatalytic
hydrogenation of lignin-derived phenol
into alkanes by using platinum supported on graphite. Electrochemistry.

[53] Shu X., Yang Q., Yao F., Zhong Y., Ren W., Chen F., Sun J., Ma Y., Fu Z., Wang D., Li X. (2019). Electrocatalytic hydrodechlorination
of 4-chlorophenol on Pd supported multi-walled carbon nanotubes particle
electrodes. Chem. Eng. J..

[54] Chen G., Wang Z. Y., Yang T., Huang D. D., Xia D. G. (2006). Electrocatalytic
Hydrogenation of 4-Chlorophenol on the Glassy Carbon Electrode Modified
by Composite Polypyrrole/Palladium Film. J.
Phys. Chem. B.

[55] May A. S., Biddinger E. J. (2020). Strategies to Control Electrochemical Hydrogenation
and Hydrogenolysis of Furfural and Minimize Undesired Side Reactions. ACS Catal..

[56] Eckelt, A. ; Eckelt, J. ; Wolf, B. Solubility of Polymers. In Encyclopedia of Polymer Science and Technology; John Wiley & Sons, Ltd, 2011.

[57] Gorbanev Y., Soriano R., O’Connell D., Chechik V. (2016). An Atmospheric Pressure
Plasma Setup to Investigate the Reactive Species Formation. J. Vis. Exp..

[58] Garg S., Yuan Y., Mortazavi M., Waite T. D. (2022). Caveats in the Use
of Tertiary Butyl Alcohol as a Probe for Hydroxyl Radical Involvement
in Conventional Ozonation and Catalytic Ozonation Processes. ACS ES&T Eng..

[59] You H., Yang Z., Lin J., Shu R., Yin T., Tian Z., Wang C., Chen Y. (2022). Hydrogenation of Lignin-derived
Phenolic Compounds over Ru/C Enhanced by Al2O3 Catalyst at Room Temperature. ChemistrySelect.

[60] Zhan Y., Di Y., Gu Z., Zhu Z., Xie C., Hu C. (2024). Catalytic
dechlorination and deep hydrogenation of 4-chlorophenol in a hydrogen-based
internal circulation reactor for efficient wastewater detoxification. J. Water Process Eng..

[61] Li A., Shen K., Chen J., Li Z., Li Y. (2017). Highly selective
hydrogenation of phenol to cyclohexanol over MOF-derived non-noble
Co-Ni@NC catalysts. Chem. Eng. Sci..

[62] Khan M. J., Wibowo A., Karim Z., Posoknistakul P., Matsagar B. M., Wu K. C., Sakdaronnarong C. (2024). Wastewater
Treatment Using Membrane Bioreactor Technologies: Removal of Phenolic
Contaminants from Oil and Coal Refineries and Pharmaceutical Industries. Polymers.

[63] Descorme C. (2017). Catalytic
wastewater treatment: Oxidation and reduction processes. Recent studies
on chlorophenols. Catal. Today.

[64] Li X. Z., Zhao Q. L., Hao X. D. (1999). Ammonium
removal from landfill leachate
by chemical precipitation. Waste Manage..

[65] Kamali M., Khodaparast Z. (2015). Review on
recent developments on pulp and paper mill
wastewater treatment. Ecotoxicol. Environ. Saf..

[66] Gu Z., Zhang Z., Ni N., Hu C., Qu J. (2022). Simultaneous
Phenol Removal and Resource Recovery from Phenolic Wastewater by Electrocatalytic
Hydrogenation. Environ. Sci. Technol..

[67] Wang Y., Jie M., Zhang H., Yang J., Xu M. (2023). High-Efficiency Mixotrophic
Denitrification for Nitrate Removal in High-Sulfate Wastewater Using
UASB Reactor. Water.

[68] Soto R., Fité C., Ramírez E., Tejero J., Cunill F. (2023). Deactivation
of macroporous ion-exchange resins by acetonitrile and inhibition
by water in the simultaneous synthesis of ethyltert-butyl ether (ETBE)
andtert-amyl ethyl ether (TAEE). React. Chem.
Eng..

[69] Wei Q., Zhang J., Luo F., Shi D., Liu Y., Liu S., Zhang Q., Sun W., Yuan J., Fan H., Wang H., Qi L., Liu G. (2022). Molecular mechanisms
through which different carbon sources affect denitrification by Thauera
linaloolentis: Electron generation, transfer, and competition. Environ. Int..

[70] Musat F., Wilkes H., Behrends A., Woebken D., Widdel F. (2010). Microbial
nitrate-dependent cyclohexane degradation coupled with anaerobic ammonium
oxidation. ISME J..

[71] Kikuchi S., Fujitani H., Ishii K., Isshiki R., Sekiguchi Y., Tsuneda S. (2023). Characterisation of bacteria representing
a novel Nitrosomonas
clade: Physiology, genomics and distribution of missing ammonia oxidizer. Environ. Microbiol. Rep..

[72] Daims H., Lebedeva E. V., Pjevac P., Han P., Herbold C., Albertsen M., Jehmlich N., Palatinszky M., Vierheilig J., Bulaev A., Kirkegaard R. H., von Bergen M., Rattei T., Bendinger B., Nielsen P. H., Wagner M. (2015). Complete nitrification by Nitrospira
bacteria. Nature.

[73] Zhang Z., Lv X., Baig S. A., Xu X. (2014). Catalytic
dechlorination of2,4-dichlorophenol
by Ni/Fe nanoparticles in the presence of humic acid: intermediate
products and some experimental parameters. J.
Exp. Nanosci..

[74] Esplugas S., Gimenez J., Contreras S., Pascual E., Rodriguez M. (2002). Comparison
of different advanced oxidation processes for phenol degradation. Water Res..

[75] D̵uričić T., Prosen H., Kravos A., Mićin S., Kalčíková G., Malinović B. N. (2023). Electrooxidation
of Phenol on Boron-doped Diamond and Mixed-metal Oxide Anodes: Process
Evaluation, Transformation By-products, and Ecotoxicity. J. Electrochem. Soc..

