# Supplementary material for: Electrocatalytic Hydrogenation of Bioinhibiting Aromatics to Bioavailable Aliphatics for Wastewater Denitrification Enhancement
Source: Environ Sci Technol. 2025 Jul 28;59(33):17640–54. doi: 10.1021/acs.est.5c00950 (PMC12755200; doi:10.1021/acs.est.5c00950)
Supplement: Supplementary file 1 [file es5c00950_si_001.pdf]

Supporting Information

Electrocatalytic Hydrogenation of Bio-inhibiting Aromatics to Bioavailable Aliphatics for Wastewater Denitrification Enhancement

Shan Shao[1], Shicong Du[1], Asma Batool[1], Yichun Lu[1], Kwun Hin Fung[1], Shuquan Huang[3], Yangxin Jin[1], Shengqin Liu[1], Qi Zhu[1], Yuhe He[1], Alicia Kyoungjin An[2], Patrick K. H. Lee[1], Jason Chun-Ho Lam[1]*

[1]School of Energy and Environment and State Key Laboratory of Marine Environmental Health, City University of Hong Kong, Kowloon Tong 999077, Hong Kong SAR, China.

[2]Department of Chemical and Biological Engineering, Hong Kong University of Science and Technology, Clear Water Bay, Kowloon 999077, Hong Kong SAR, China.

[3]Faculty of Chemical Engineering, Kunming University of Science and Technology, Kunming, Yunnan 650500 China

*Email: jason.lam@cityu.edu.hk

Summary: 15 pages, 2 texts, 10 tables, 13 figures, 6 references.

Text S1. The standard potential conversion

All the potentials in this study were calibrated with a reversible hydrogen electrode (RHE) using the Nernst equation as follows:

$$E_{RHE} = E_{Ag/AgCl} + 0.197\ V + 0.059\ V \times pH$$

Where E_{RHE} represents the converted potential vs. RHE, $E_{Ag/AgCl}$ is the obtained potential vs. Ag/AgCl, and 0.197 V is the electrode potential of Ag/AgCl at ambient temperature (25 °C).

Text S2. Inductively Coupled Plasma Optical Emission Spectrometry (ICP-OES)

Inductively Coupled Plasma Optical Emission Spectrometry (ICP-OES) measurements were performed on an ICP-OES Optima 8000 spectrometer. The emission intensity of dissolved Ru ions was measured at 240.272 nm. All reaction samples were diluted 10 times with 2% HNO_3 before the ICP-MS analysis.

Table S1. Summary table of the main acronyms used in the text

Acronyms	Descriptions	Acronyms	Descriptions
ECH	Electrochemical hydrogenation	CV	Cyclic voltammetry
AOPs	Advanced oxidative protocols	ICP-OES	Inductively coupled plasma optical emission spectrometry
ROS	Reactive oxygen species	PBS	Phosphate buffer solution
ET	Electron transfer	SA	Sodium alginate
SBR	Sequencing batch reactor	SAS	Sodium dodecyl sulfate
ACC	Activated carbon cloth	HA	Humic acid
SEM	Scanning electron microscopy	ACN	Acetonitrile
EDX	Energy-dispersion X-ray	5-HMF	5-hydroxymethylfurfural
HRTEM	High-resolution transmission electron microscopy	GC-MS	Gas chromatography-mass spectrometry
XRD	X-ray diffraction	PHE	Phenol
4-CP	4-chlorophenol	CYL	Cyclohexanol
HER	Hydrogen evolution reaction	BLK	Blank
WWTP	Wastewater treatment plant	NO3	Nitrate-only control
FE	Faraday efficiency	WRST	Wilcoxon rank-sum test
EPR	Electron paramagnetic resonance	ASVs	Amplicon sequence variants
DMPO	5,5-dimethyl-1-pyrroline N-oxide	EO	Electrooxidation
TBA	Tert-butyl alcohol	DRE	Denitrification rate enhancement

Table S2. The pH monitoring of the electrolyte before and after the reaction.

Categories	Solutions	pH	Equal volume mixture
Without Reactant	0.5 M PBS (before reaction)	7	Equal volume mixture
	0.5 M PBS (after 1h reaction) Anolyte	2.6	**6.97**
	0.5 M PBS (after 1h reaction) Catholyte	11.8	
	0.5 M PBS (after 2h reaction) Anolyte	1.5	**6.71**
	0.5 M PBS (after 2h reaction) Catholyte	13.14	
With Reactant	20 mM Phenol+0.5 M PBS (before reaction)	7.15	Equal volume mixture
	20 mM Phenol+0.5 M PBS (after 1h reaction) Anolyte	2.6	**7.01**
	20 mM Phenol+0.5 M PBS (after 1h reaction) Catholyte	11.73	
	20 mM Phenol+0.5 M PBS (after 2h reaction) Anolyte	1.5	**6.82**
	20 mM Phenol+0.5 M PBS (after 2h reaction) Catholyte	12.9	

Table S3. Aliphatic products transformation after 5-day denitrification process

**Entry**	**Aliphatic products**	**The product amount change after denitrification (mM)**
**1**	Cyclohexanol	-2.88
	2-Methoxycyclohexanol	-3.88
**2**	Cyclohexanol	-11.84
	Cyclohexanone	+0.12
**3**	Cyclohexanol	-10.1
	Cyclohexanone	-0.15
**4**	Benzyl alcohol	-11.97
**5**	2,5-Bis(hydroxymethyl)furan	-9.51
**6**	4-Aminobenzyl alcohol	-9.84
**7**	Cyclohexanol	-10.07
	Cyclohexanone	+0.02
**8**	Cyclohexanol	-3.13
	Cyclohexanone	+0.07
	Phenol	-0.08
**9**	Cyclohexanol	-9.63
	Cyclohexanone	+0.06
	Phenol	-0.84
**10**	Cyclohexanol	-9.51
	Cyclohexanone	+0.66
	Phenol	-1.66
**11**	Cyclohexanol	-2.57
	Cyclohexanone	+1.35
	Phenol	-0.15
**12**	1-Cyclohexylethan-1-ol	-11.01
	1-Cyclohexylethan-1-one	+0.55

Table S4. Conditions for the BLK, NO3, 4-CP, PHE, and CYL treatment

Additives / Name	KNO_3	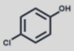	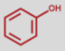	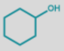
BLK				
NO3	✓			
4CP	✓	✓		
PHE	✓		✓	
CYL	✓			✓

Table S5. Pairwise comparisons using pairwise Wilcoxon rank sum test.

Richness	**4CP**	**BLK**	**CYL**	**NO3**
**BLK**	1	-	-	-
**CYL**	1	1	-	-
**NO3**	0.017	0.017	0.017	-
**PHE**	0.155	0.112	0.155	0.693
**Pielou' *J* Evenness**	**4CP**	**BLK**	**CYL**	**NO3**
**BLK**	0.3869	-	-	-
**CYL**	**0.0072**	0.133	-	-
**NO3**	0.7768	0.8182	0.1082	-
**PHE**	**0.0108**	**0.0072**	**0.0072**	0.1082
Thauera	**4CP**	**BLK**	**CYL**	**NO3**
**BLK**	**0.011**	-	-	-
**CYL**	**0.043**	0.818	-	-
**NO3**	0.438	**0.011**	**0.03**	-
**PHE**	**0.022**	0.093	0.387	**0.014**
Nitrosomonas	**4CP**	**BLK**	**CYL**	**NO3**
**BLK**	**0.0036**	-	-	-
**CYL**	**0.0036**	0.1034	-	-
**NO3**	**0.0036**	**0.0216**	0.8182	-
**PHE**	0.1034	**0.0036**	**0.0036**	**0.0036**

Table S6. Quality of the collected wastewater samples.

Parameter	Unit	Landfill leachate	Municipal wastewater
Turbidity	NTU	900	1103
pH	/	8.22	7.35
TOC	mg/L	2054	140.72
COD	mg/L	760	232
Conductivity	mS/cm	23.00	17.56
TDS	g/L	11.40	9.35
Ammonium-N	mg/L	13.34	516.63
Nitrate-N	mg/L	3.76	n.d.
Nitrite-N	mg/L	n.d.	n.d.
Chlorides	mg/L	452.57	717.77
Sulfate	mg/L	76.30	517.98
Dissolved oxygen	mg/L	2.40	1.60

TOC: Total organic carbon; COD: Chemical oxygen demand; TDS: Total dissolved solids.
* n.d.: not detected.

Table S7. The ICP-OES result of the electrolyte after the reaction.

Mean Data:		
Analyte	Conc. Units	Std.Dev.
Ru 240.272	0.0005 mg/L	0.00035

Table S8. Parameter selection for the cost-effectiveness analysis.

Parameter	Value range	Unit	Reference and note
The volume of wastewater treated	100,000	gallon/day	Based on the traditional denitrification cost estimation.
Operational lifespan of electrode	100	day	
Operational lifespan of Nafion 117	100	day	**[1]**
Ru price	14.95	\$/kg	**[2]**
Ru deposit amount	0.01	kg/m^2	Based on the experimental result, 1.6 mg Ru was deposited on the ACC surface (1.5 cm^2) after electrodeposition process.
Carbon cloth price	682	\$/m^2	40 × 40 cm Carbon cloth price: 800 RMB ≈ 109.12 USD
Nafion 117 price	1400-2200	\$/m^2	**[3]**
Electrode area	1.5	m^2	
Nafion 117 area	1	m^2	
Traditional denitrification cost	1.23-3.42	\$/1000 gallon	**[4]**
The NO_3-N concentration of the untreated wastewater	20	mg/L	$C(NO_3-N, untreated)$
The target NO_3-N concentration of the effluent discharge	10	mg/L	$C(NO_3-N, discharge)$, the environmental target NO_3-N concentration in discharged water (< 10 mg/L)**[5]**
The NO_3-N concentration to be treated	10	mg/L	$C(NO_3-N, treated)$
Traditional denitrification rate	10	mg/L/day	
The denitrification rate enhancement (DRE)	80.7	%	Based on the 4-chlorophenol (dissolved in wastewater, Figure 3c) result.
Methanol needed	1.4:1	CH_3OH-C: NO_3-N ratio	Methanol is one of the most widely used external carbon sources due to its effectiveness and low cost.**[5]**
Methanol price	2.5-3.0	\$/gallon	**[5]**

Table S9. Summary of mathematical formula used for cost-effectiveness analysis.

Description	Mathematical Formula
Energy Consumption (kWh kg_{phenol}^{-1})	$= \frac{average\ cell\ potential\ (V) \times applied\ current\ (A) \times reaction\ time\ (h)}{Mass\ of\ consumed\ phenol\ (kg)}$
Energy Consumption (kWh m^{-3})	$= \frac{average\ cell\ potential\ (V) \times applied\ current\ (A) \times reaction\ time\ (h)}{solution\ volumn\ (m^3)}$
Electricity Cost ($ m^{-3})	$= energy\ consumption\ (kWh\ m^{-3}) \times electricity\ price\ (\$\ kWh^{-1})$
Electrode Cost ($ day^{-1})	$= \frac{\left(Ru\ cost\ \left(\frac{\$}{m^2}\right) + carbon\ cloth\ cost\ \left(\frac{\$}{m^2}\right)\right) \times electrode\ area\ (m^2)}{electrode\ lifespan\ (day)}$
Nafion 117 Cost ($ day^{-1})	$= \frac{Nafion\ price\ \left(\frac{\$}{m^2}\right) \times membrane\ area\ (m^2)}{membrane\ lifespan\ (day)}$
Traditional Denitrification Cost ($ day^{-1})	$= average\ spending\ (\$\ gallon^{-1}) \times wastewater\ volumn\ (gallon\ day^{-1})$
Treated NO_3-N Concentration (mg/L)	$= C(NO_3 - N, untreated)\ (mg/L) - C(NO_3 - N, discharge)\ (mg/L)$
Traditional Denitrification rate $(mg/L\ day^{-1})$	$= \frac{C(NO_3 - N, treated)\ (mg/L)}{treatment\ time\ (day)}$
Pre-ECH denitrification time (t, day)	$DRE(\%) = \frac{\frac{C(NO_3 - N, treated)\ \left(\frac{mg}{L}\right)}{t\ (day)} - R_{traditional}\ \left(\frac{mg}{L} day^{-1}\right)}{R_{traditional}\ (mg/L\ day^{-1})}$
Methanol cost ($ day^{-1})	$= \frac{Methanol\ needed(gallon) \times Methanol\ price\ (\$\ gallon^{-1})}{treatment\ time\ (day)}$

Table S10. Summary of the cost-effectiveness analysis

Classifications	Unit	Amount	Total
**ECH cell**			
Electrode	$\$ \ day^{-1}$	10.23	
Nafion 117	$\$ \ day^{-1}$	14-22	24.23-32.23
**Traditional denitrification process**			
Cost	$\$ \ day^{-1}$	123-342	123-342
**Methanol needed (used as carbon source)**			
Cost	$\$ \ day^{-1}$	11.78-14.13	11.78-14.13
**Pre-ECH time saving**			
1-day treatment	day	0.45	0.45
**Pre-ECH cost saving**			
1-day treatment	$\$ \ day^{-1}$	23.12-129.67	**23.12-129.67**

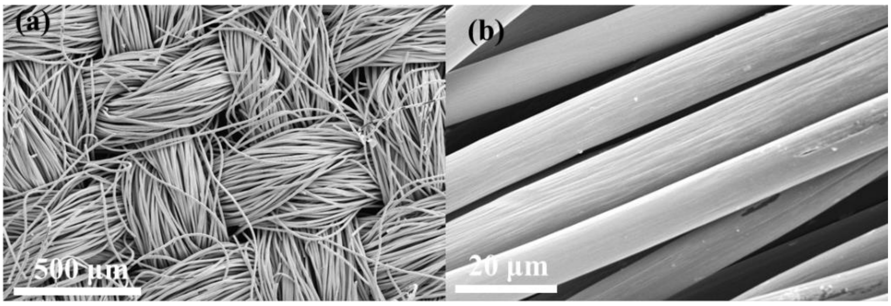

Figure S1. SEM images of Ru/ACC.

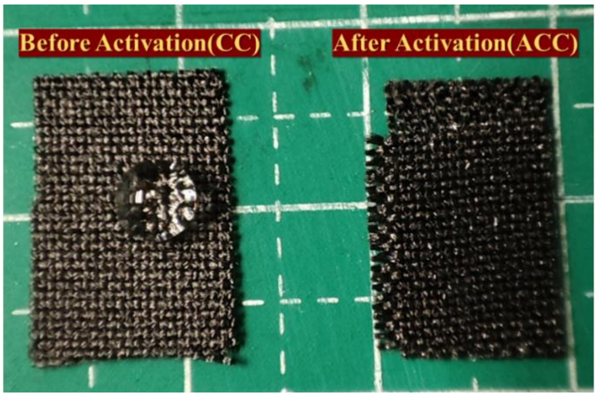

Figure S2. Hydrophilicity comparison of before and after activation process.

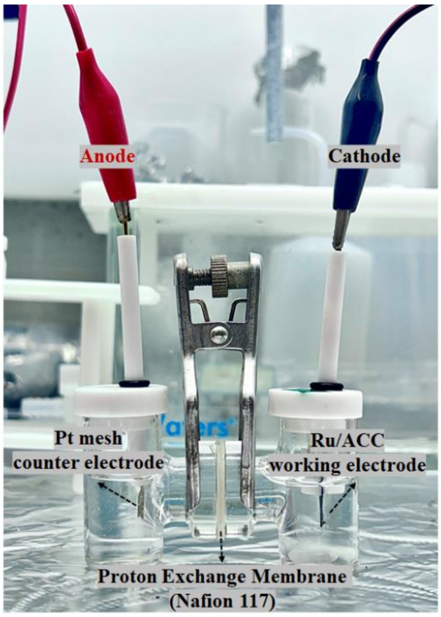

Figure S3. H-type electrochemical cell (H-cell) setup.

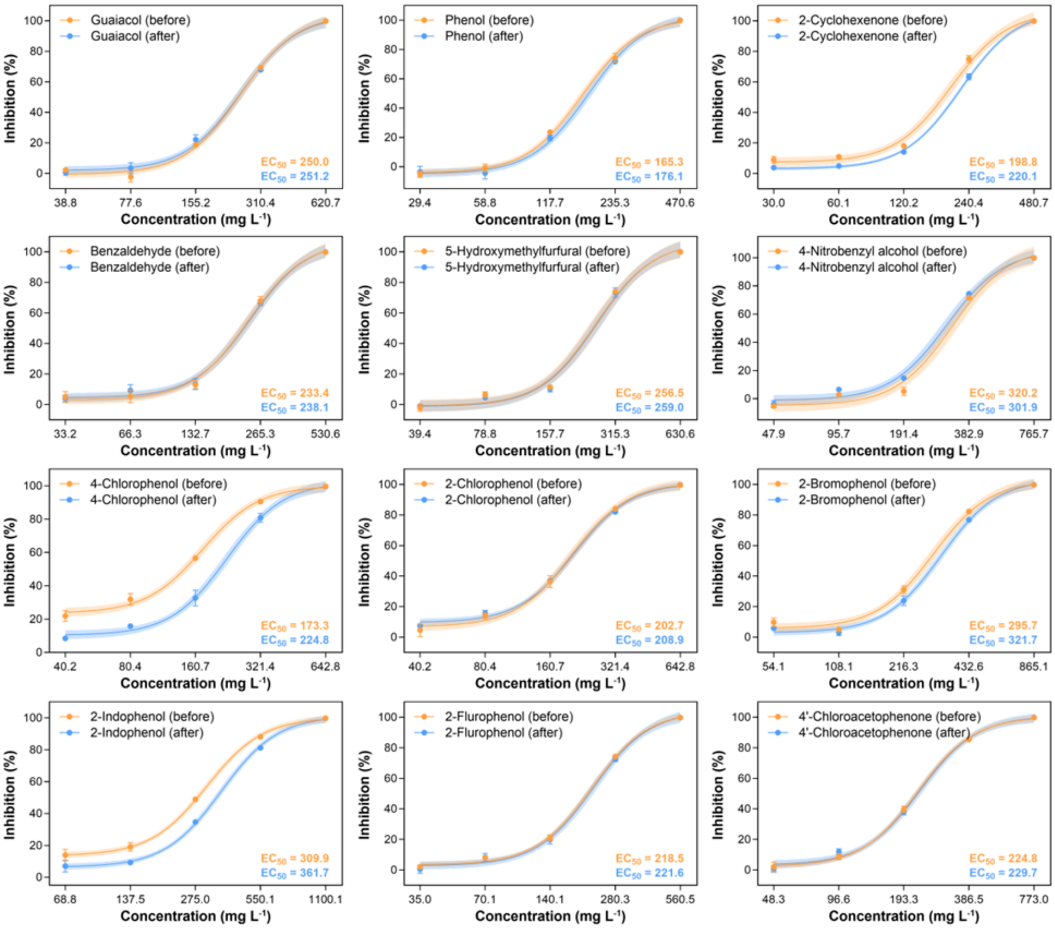

Figure S4. Concentration-response curves for EC_{50} measurements.

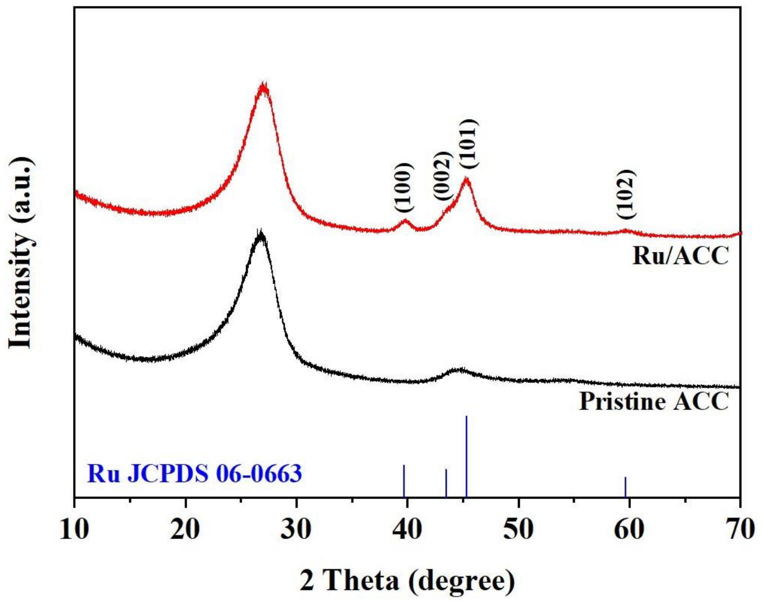

Figure S5. XRD images of Ru/ACC.

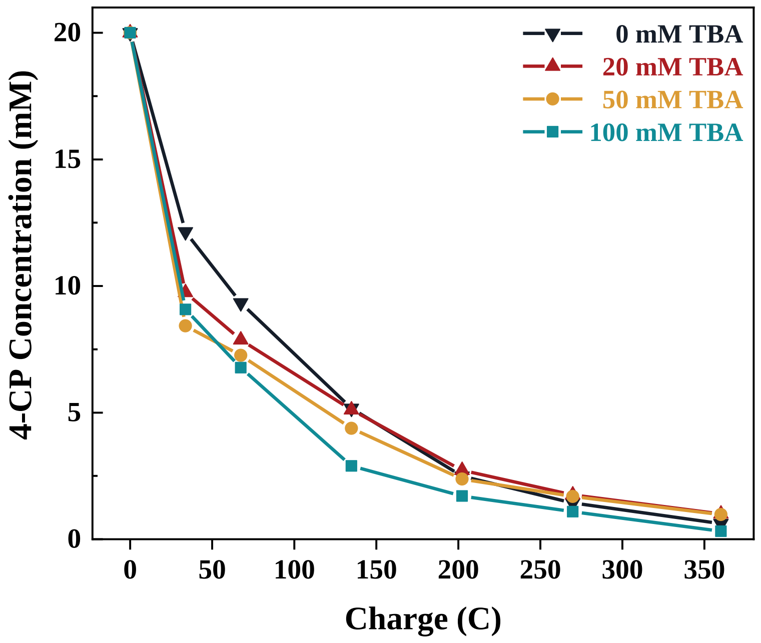

Figure S6. Removal performance of 4-CP in the presence of different concentrations of TBA.

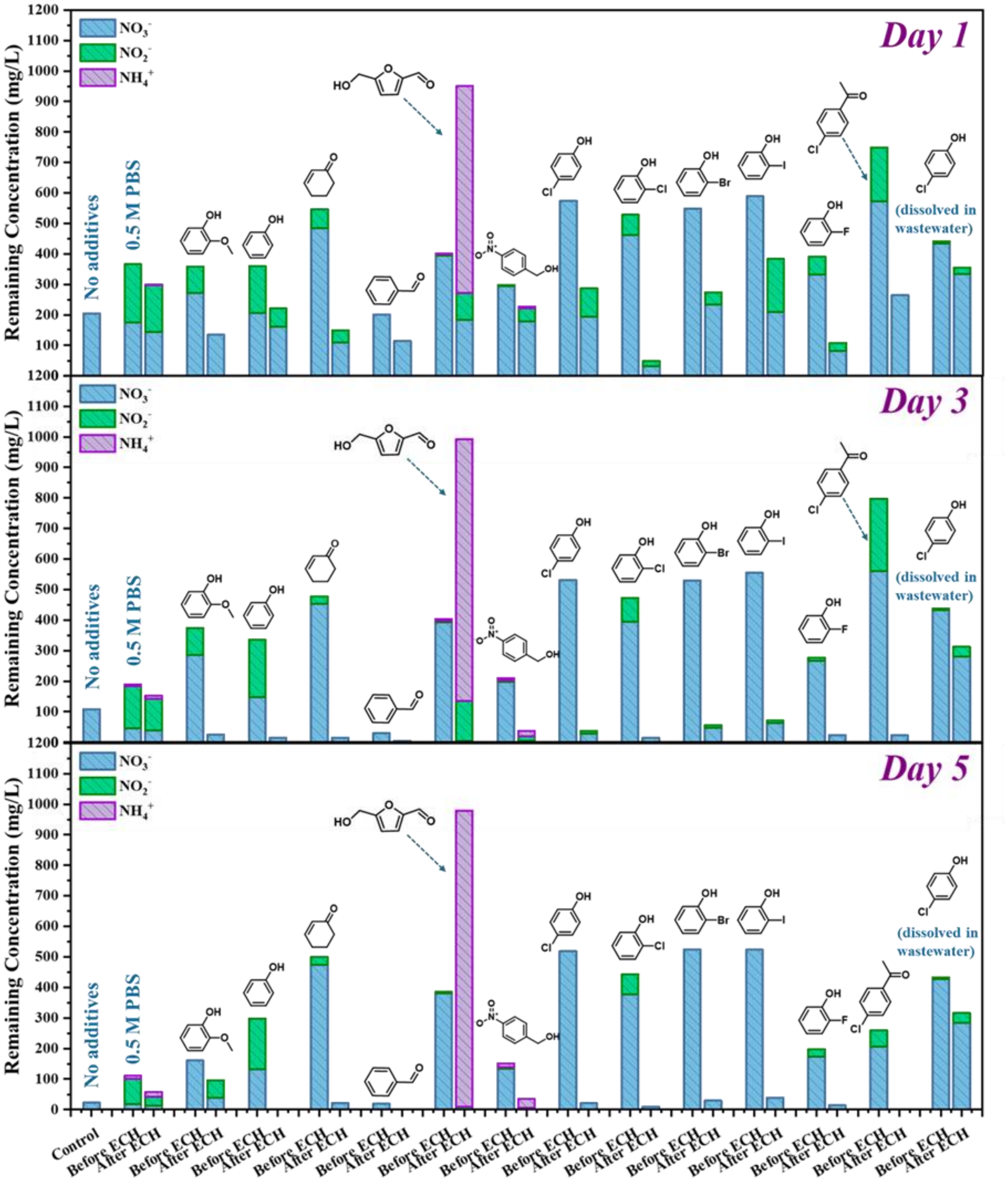

Figure S7. Nitrate, nitrite, ammonia nitrogen, and total nitrogen information after 1-day, 3-day, and 5-day incubation.

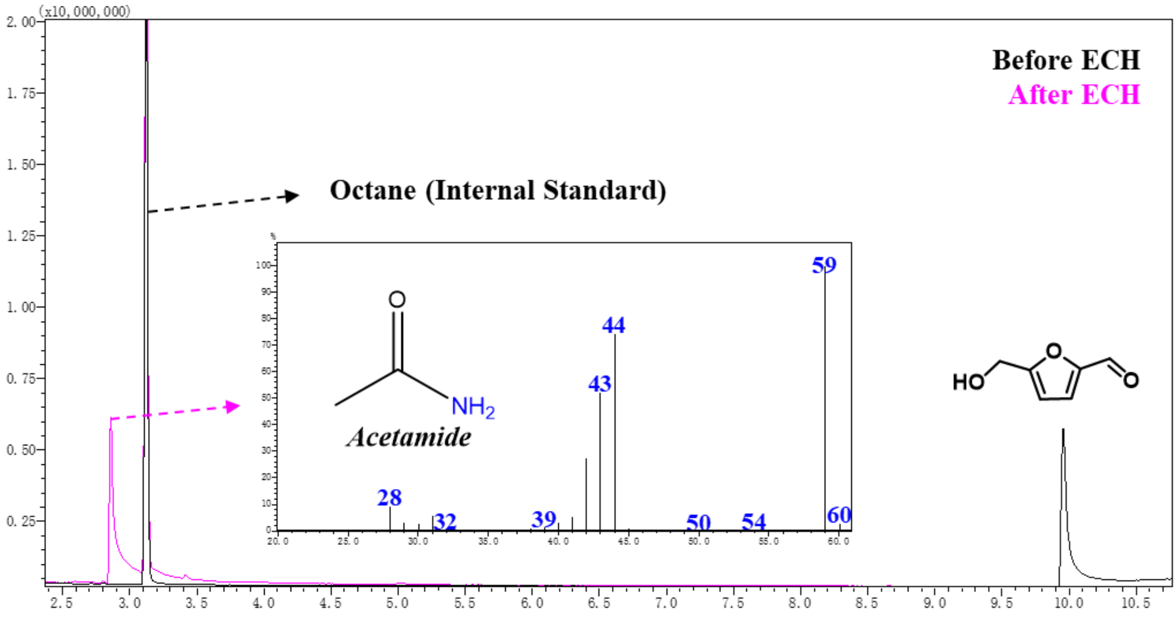

Figure S8. The gas chromatography-mass spectrometry (GC-MS) results of 5-Hydroxymethylfurfural before and after ECH.

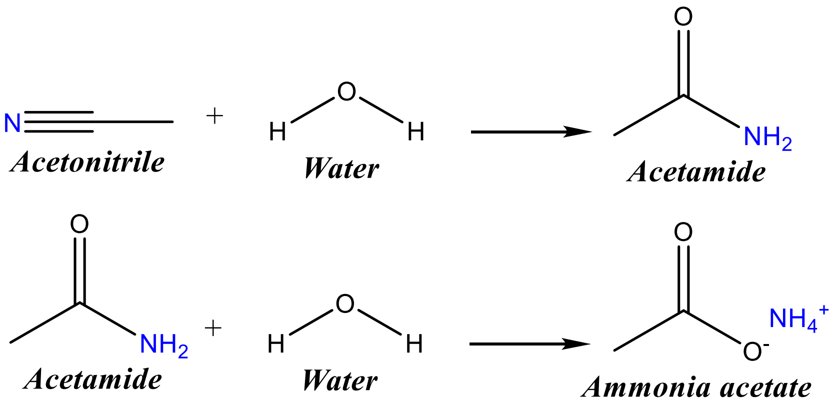

Figure S9. Acetonitrile direct hydrolysis reactions.**[6]**

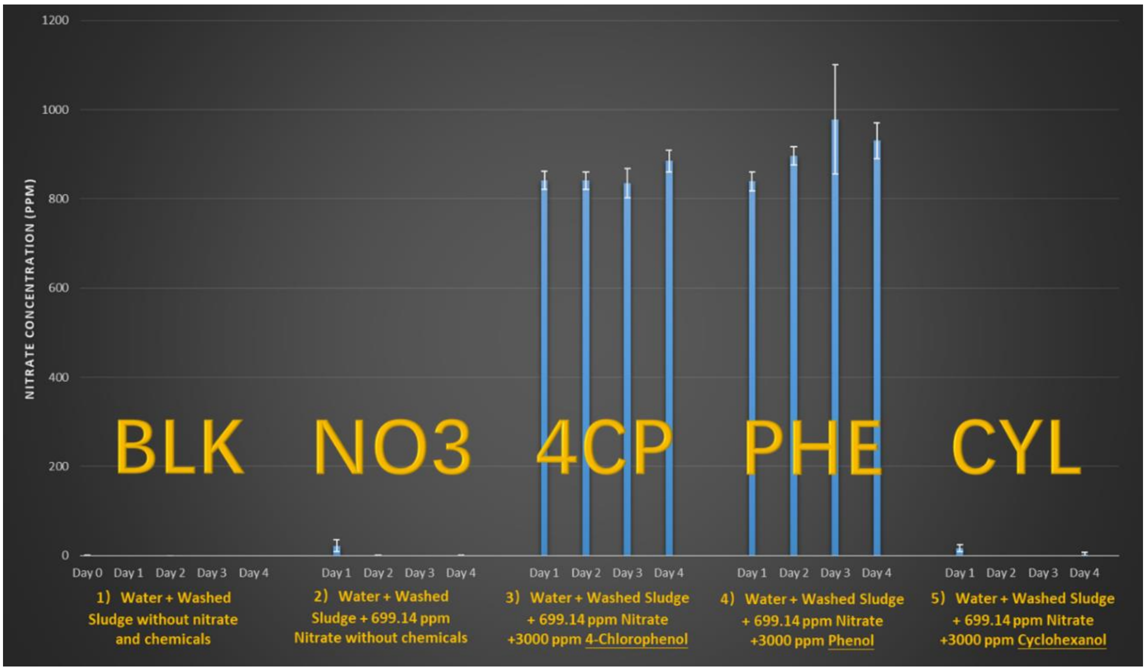

Figure S10. Monitoring of nitrate concentration changes over four days.

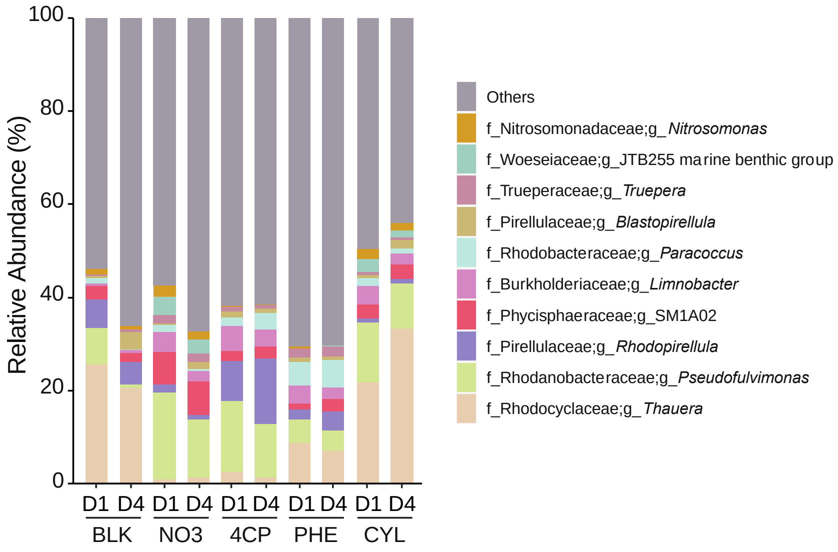

Figure S11. The average relative abundance of the top 10 genera in bacterial communities in day 1 (D1) and day 4 (D4) across five treatments.

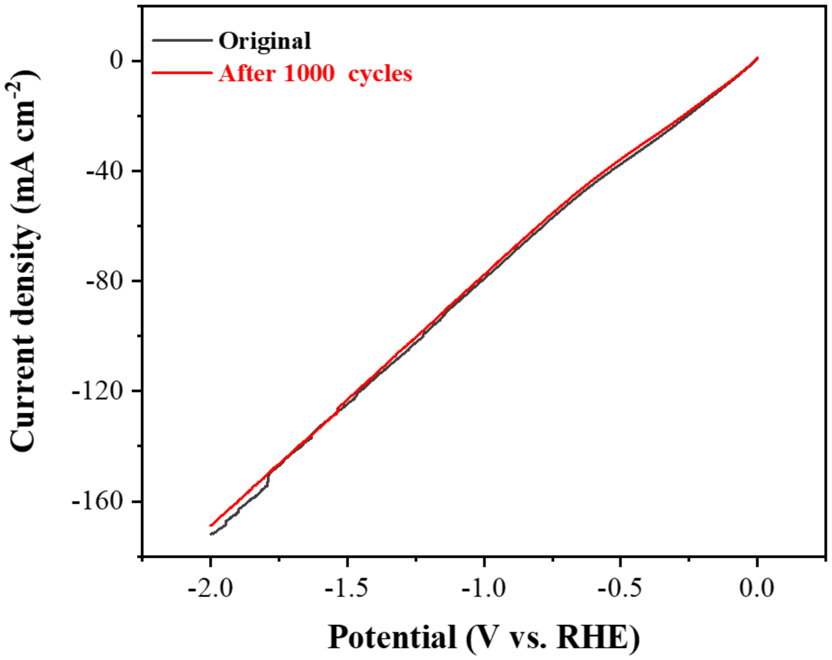

Figure S12. Polarization curves of Ru/ACC before and after 1000 CV cycles.

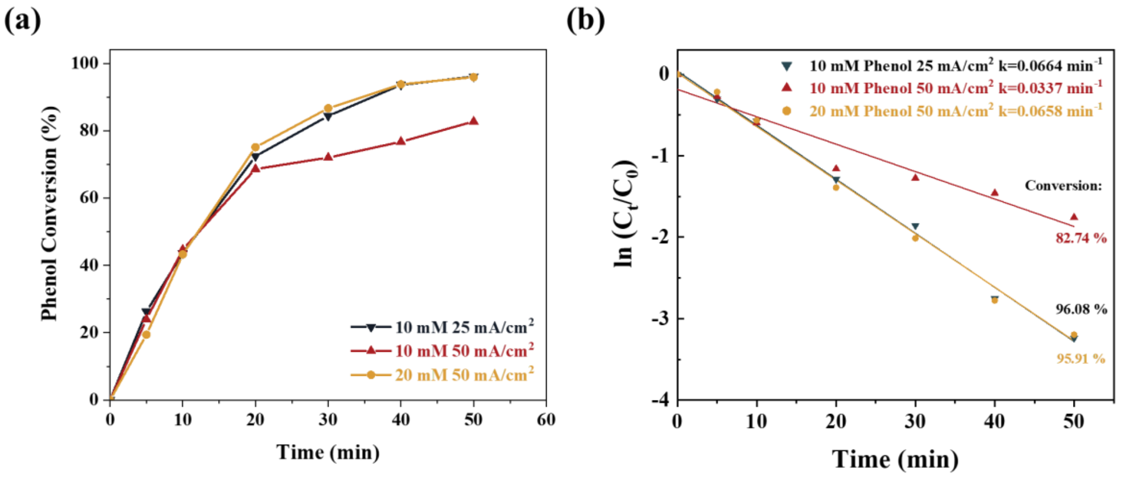

Figure S13. (a) Removal performance of phenol in the presence of different comcentration and current density. (b) Pseudo-first-order kinetic fitting curve, ln $(C_t/C_0) = -kt$, of ECH of 20 mM Phenol in 0.5 M PBS (pH 7) at 80 C° and 50 mA/cm^2.

Reference

1. Seema, S., et al., *Ageing Effect of Proton Exchange Membrane Nafion 117 in Different Solutions*. Nanotechnology Perceptions, 2024. **20**(8).

2. *Daily Metal Prices*. 2025 [cited 2025 Jan 14]; Available from: https://www.dailymetalprice.com/metalprices.php?c=ru&u=kg&d=1.

3. Hernández-Flores, G., H.M. Poggi-Varaldo, and O. Solorza-Feria, *Comparison of alternative membranes to replace high cost Nafion ones in microbial fuel cells.* International Journal of Hydrogen Energy, 2016. **41**(48): p. 23354-23362.

4. *Survey Examines Wastewater Treatment Costs*, in *Water Technology*. 2011.

5. Coletti, L., et al., *Wood chip denitrification bioreactors can reduce nitrate in tile drainage.* California Agriculture, 2017. **71**(1): p. 41-47.

6. Soto, R., et al., *Deactivation of macroporous ion-exchange resins by acetonitrile and inhibition by water in the simultaneous synthesis of ethyltert-butyl ether (ETBE) andtert-amyl ethyl ether (TAEE).* Reaction Chemistry & Engineering, 2023. **8**(2): p. 389-401.
